# Peripubertal Bisphenol A Exposure Imparts Detrimental Age-Related Changes in Body Composition, Cognition, and Hydrogen Sulfide Production Capacities

**DOI:** 10.1089/ars.2020.8226

**Published:** 2022-06-03

**Authors:** Jie Yang, Christopher Link, Yoko O. Henderson, Nazmin Bithi, Christopher Hine

**Affiliations:** Department of Cardiovascular and Metabolic Sciences, Cleveland Clinic Lerner Research Institute, Cleveland, Ohio, USA.

**Keywords:** bisphenol A (BPA), endocrine disruption, peripubertal, cognition, H_2_S, aging

## Abstract

**Aims:**

Peripubertal endocrine disruption has immediate and lifelong consequences on health, cognition, and lifespan. Disruption comes from dietary, environmental, and pharmaceutical sources. The plasticizer Bisphenol A (BPA) is one such endocrine disrupting chemical. However, it is unclear whether peripubertal BPA exposure incites long-lasting physiological, neuro-cognitive, and/or longevity-related metabolic impairments. Catabolism of cysteine *via* transsulfuration enzymes produces hydrogen sulfide (H_2_S), a redox-modulating gasotransmitter causative to endocrine and metabolic homeostasis and improved cognitive function with age. As thyroid hormone (TH) regulates hepatic H_2_S production and BPA is a TH receptor antagonist, we hypothesized that BPA exposure during peripubertal development impairs metabolic and neuro-cognitive/behavioral endpoints in aged mice, in part, due to altered peripheral and/or localized H_2_S production and redox status.

**Results:**

To test this, male C57BL/6J mice at 5 weeks of age were orally exposed daily for 5 weeks to 250 μg BPA/kg, defined as low dose group (LD BPA), or 250 mg BPA/kg, defined as high dose group (HD BPA). Both LD and HD BPA exposure decreased lean mass and increased fat mass accompanied by decreased serum total TH at advanced ages. In addition, LD BPA had an anxiogenic effect whereas HD BPA caused cognitive deficits. Notably, HD BPA disrupted tissue-specific H_2_S production capacities and/or protein persulfidation, with the former negatively correlated with memory deficits and oxidative stress.

**Innovation and Conclusion:**

These findings provide a potential mechanism of action for acute and long-term health impacts of BPA-induced peripubertal endocrine disruption and bolster the need for improved monitoring and limitation of adolescent BPA exposure. *Antioxid. Redox Signal.* 36, 1246–1267.

**Figure:**
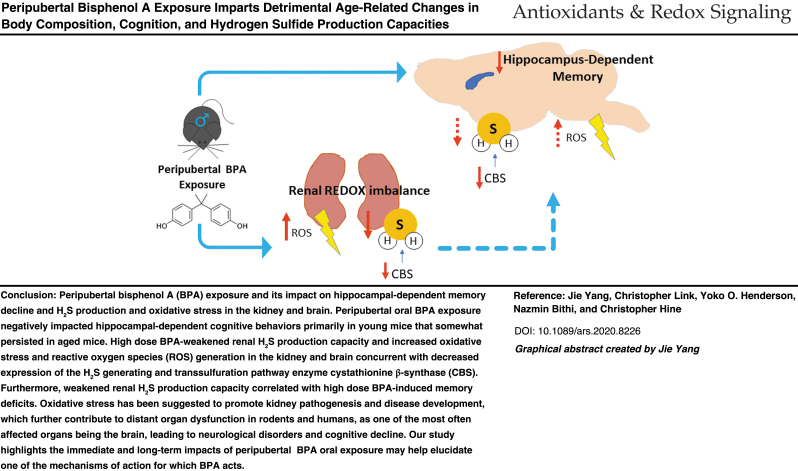
(*Color images are available online*).

## Introduction

Abnormalities in endocrine signaling during early life and pubertal development result in immediate and long-lasting changes in metabolism, resiliency, cancer susceptibility, and other health-related endpoints (67). This paradigm is termed the Developmental Origins of Health and Disease (DOHAD) (20). Adolescents during this critical stage are voluntarily and involuntarily exposed to endocrine disrupting chemicals (EDCs), with one of these EDCs being Bisphenol A (BPA) (36). A 44-fold higher BPA level in U.S. adults was recently reported by using a direct detection method (17) compared with traditional indirect methods (36), highlighting a great underestimation of BPA levels and its unrecognized effect on health. Strikingly, in a representative cohort study in U.S. adults, high BPA exposure was associated with increased risk of all-cause mortality (4).

**Innovation**

Our results can be used to model and predict potential acute and long term dangers unknowingly imposed on children as they grow into adulthood and later life. Likewise, the results can be used as a conceptual tool to develop targeted interventions to address the BPA-triggered health issues.

Thus, BPA negatively affects the lifespan and health span while accelerating the onset of aging-related diseases. As BPA exposure in children has become a major health concern in the past 50 years due to dramatic increases in plastics production, incorporation into food packaging, and inability to properly manage its waste (8, 18), it is paramount that we fully understand the immediate and long-term molecular and physiological impacts that this chemical induces.

BPA is an estrogen receptor (ER) agonist (12) and it also exerts thyroid hormone (TH) receptor antagonizing effects (41) with its lowest-observed-adverse-effect-level (LOAEL) evaluated at 50 mg/kg body weight (b.w.) and the reference dose set at 50 μg/kg b.w. by the U.S. Environmental Protection Agency (U.S. EPA) (64a). Interestingly, a number of studies in rodents, including the Consortium Linking Academic and Regulatory Insights on BPA Toxicity (CLARITY-BPA) risk assessment program (49), show that BPA disrupts hormonal activities, sexual dimorphism, and neurobehavioral development at doses that are several orders of magnitude below the safe level prescribed in initial regulatory studies (40, 49, 65). Likewise, inconsistencies exist with regard to strain, species, and exposure time sensitivity to low-dose effects of BPA, which necessitates a better physiologic and mechanistic understanding of immediate and long-term impacts of BPA.

Maximal disruption of endocrine homeostasis by EDCs is believed to be during fetal and early postnatal life (45). Epidemiological studies implicate that BPA exposure during early life (prenatal, postnatal, and early childhood) is associated with behavior defects in children (7, 22, 52). However, studies examining BPA exposure during peripuberty are scarce, which is surprising as this period is crucial for organ system maturation and cognition development (9). Hence, we sought to determine acute and aging-related physiological, cognitive, and redox-related changes imparted by peripubertal BPA exposure.

Hydrogen sulfide (H_2_S) is recognized as a redox-modifying gasotransmitter that impacts numerous biochemical and cellular processes (68, 74). H_2_S improves insulin sensitivity and responses to glucose (70), facilitates hippocampal long-term potentiation (32), delays cognitive decline in animal models of neurodegeneration (19, 48), and alters many physiological and aging-related endpoints with clinical relevance (68). Tissue-specific regulation of endogenous H_2_S production is partially through hormonal activities, including estrogen (75) and TH (23). Likewise, H_2_S itself can be considered an endocrine and/or paracrine factor due to its stimulatory effects on angiogenesis (63) and its reciprocal regulation of insulin (73), insulin-like growth factor 1 (IGF-1) (23, 58), and TH (23).

Therefore, in this study, we examined acute and life-long effects of peripubertal BPA exposure on physiology and neurobehavioral development, specifically anxiety and cognition in male mice. We selected males, as BPA exposure is more likely to affect behavior and neurological development (22, 52) and growth in peripubertal male humans (69). In addition, we sought to further the idea of BPA as an EDC with the potential to alter tissue-specific H_2_S generation and oxidative stress. We found that peripubertal BPA exposure impaired body composition and glucose metabolism concurrent with dose-specific oxidative stress in the brain and anxiety-like and cognitive defects, to which the latter negatively correlated with renal H_2_S production capacity and brain cystathionine β-synthase (CBS) expression.

## Results

### Peripubertal BPA exposure and its impact on acute and longitudinal weight, body mass composition, and metabolic function

**FIG. 1.**
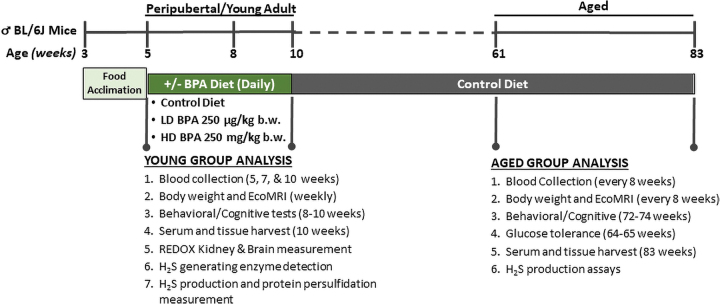
**Experimental design for testing multicomponent immediate and long-term outcomes of peripubertal BPA exposure.** Male C57BL/6J mice at 5 weeks of age were randomly assigned to one of three diets: (i) Control diet containing no added BPA, (ii) diet with 250 μg BPA/kg b.w. per day defined as the LD BPA group, and (iii) diet with 250 mg BPA/kg b.w per day as the HD BPA group. Mice were maintained on these ± BPA-containing diets *ad libitum* for 5 weeks and analyzed for changes in weight, body composition, behavioral/cognitive abilities, endocrine and metabolic markers, oxidative stress, H_2_S production and signaling, and endpoint analysis on sampled tissues between 8 and 10 weeks of age (referred to as the Young Group Analysis) or between 61 and 83 weeks of age (referred to as the Aged Group Analysis). For the Aged Group Analysis, the mice were all placed back on the non-BPA containing control diet for the remainder of the study after the initial 5-week peripubertal experimental diet. BPA, bisphenol A; b.w., body weight; H_2_S, hydrogen sulfide; HD, high dose; LD, low dose. Color images are available online.

The overarching experimental design is depicted in Figure 1. Briefly, male C57BL/6J mice at 5 weeks of age were randomly assigned to one of three diets: (i) Control diet without BPA, (ii) 250 μg BPA/kg b.w per day defined as the low-dose BPA group (LD BPA), and (iii) 250 mg BPA/kg b.w. per day as the high-dose BPA group (HD BPA). Mice were maintained on these diets *ad libitum* for 5 weeks and analyzed for changes in weight, body composition, behavioral/cognitive abilities, endocrine and metabolic markers, and endpoint analysis on sampled tissues between 8 and 10 weeks of age (referred to as the Young Group Analysis) or between 61 and 83 weeks of age (referred to as the Aged Group Analysis). It is important to note all Aged Group mice were placed on control diet without BPA for the remainder of the study post the initial peripubertal 5 weeks experimental diet.

**FIG. 2.**
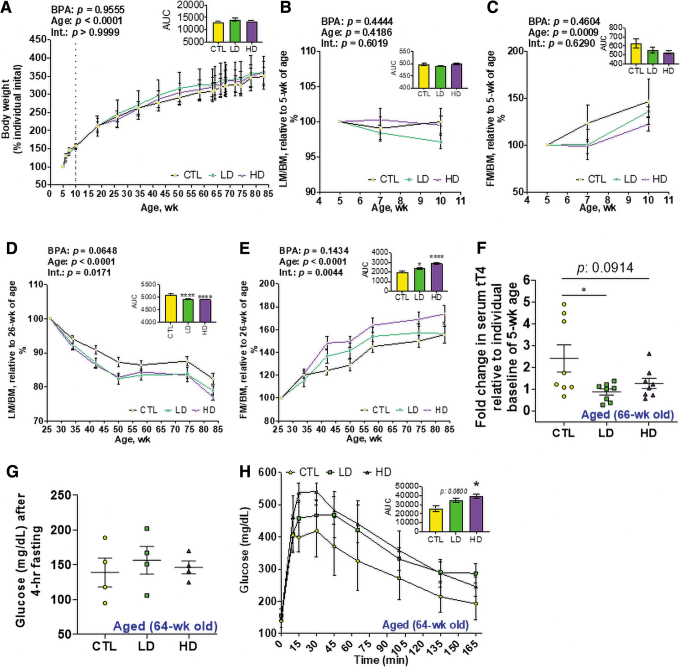
**Peripubertal BPA exposure alters long-term BM composition and metabolic function.** Body weight and BM composition from the beginning of BPA exposure at 5 weeks of age until the study completion at 83 weeks of age were analyzed. **(A)** Body weights were collected at ages as indicated and presented as a percentage to the animal's initial weight starting at 5 weeks of age. *n* = 7–8/group. **(B, C)** LM **(B)** and FM **(C)** of the Young Group animals during BPA experimental diets at 5, 7, and 10 weeks of age are presented as the percent total body weight (LM/BM and FM/BM, respectively) relative to the ratio at 5 weeks of age, *n* = 5/group. **(D, E)** LM **(D)** and FM **(E)** of the Aged Group animals at indicated ages are presented as the percent to the total body weight relative to their ratio at 26 weeks of age, *n* = 7–8/group. **(F)** Serum tT4 assayed in the Aged Group at 66 weeks and presented as the fold change relative to the individual animal baseline levels at 5 weeks of age, *n* = 8/group. **(G)** Fasting blood glucose level in the Aged Group animals at 64–65 weeks of age, *n* = 4/group. **(H)** GTT for the Aged Group animals at 64–65 weeks of age. *n* = 4/group. The AUC was calculated for each dose-defined group and depicted as the in-set figure. *Asterisk* indicates the significance of the difference *versus* the Control diet fed group; *p < 0.05 and ****p < 0.0001. All data were presented as mean ± SEM. See also Supplementary Figure S2. AUC, area-under-the-curve; BM, body mass; FM, fat mass; GTT, glucose tolerance test; LM, lean mass; SEM, standard error of the mean; tT4, total thyroxine (thyroid hormone). Color images are available online.

Growth curves, including body weight and body mass (BM) composition from the beginning of BPA exposure at 5 weeks of age until the study completion at 83 weeks of age, are shown in Figure 2. No differences in food intake between the three groups were detected during the 5-week peripubertal period (Supplementary Fig. S2A) or at age 68 weeks (Supplementary Fig. S2B). No changes in body weight were observed due to BPA exposure (Fig. 2A). Likewise, BM composition analysis determined by EchoMRI showed that the percent lean and fat mass (FM) were not changed during the 5-week peripubertal BPA exposure (Fig. 2B, C). However, both LD and HD BPA-exposed mice had decreased lean mass (LM) (Fig. 2D) and increased FM (Fig. 2E) compared with control animals when measured between 26 and 83 weeks of age.

Alterations in BM composition, particularly augmentation of FM in later life, can be driven by changes in endocrine and/or metabolic parameters (10, 42, 64). As BPA is an EDC (12, 41), we examined circulating endocrine factors regulating growth and BM composition. Serum IGF-1 and total thyroxine (TH) (tT4) were not affected by the 5 weeks of acute BPA exposure at the end of the peripubertal period (Supplementary Fig. S2C, D), indicating that the doses used were not so extreme as to impact these two factors. However, when animals reached 66 weeks of age, tT4 (Fig. 2F), but not IGF-1 (Supplementary Fig. S2E), was decreased in both LD and HD BPA-exposed groups. Further, baseline fasting glucose levels were not changed as a function of BPA exposure (Fig. 2G), whereas glucose tolerance/handling was detrimentally impacted with increasing BPA exposure in 64-week-old males (Fig. 2H). Therefore, peripubertal BPA exposure did not have an immediate impact on BM composition, serum IGF-1, or serum tT4, but it did alter later life BM composition possibly through disrupted TH signaling and/or impaired glucose metabolism.

### Peripubertal LD BPA exposure impairs locomotor activity and anxiety

**FIG. 3.**
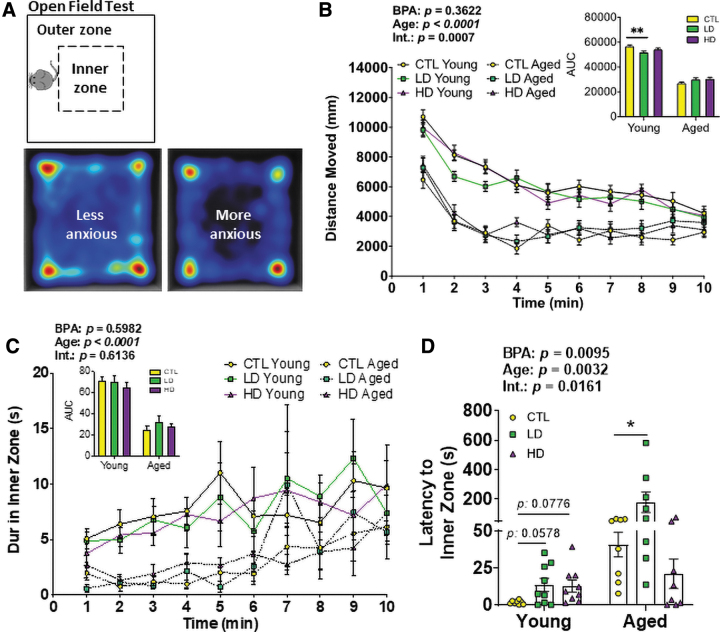
**LD BPA exposure negatively impacts locomotor activity and anxiety. (A)** Experimental design of the OF test. The test was performed in a *square open box* as shown in the *upper subpanel*, and each animal was released from the same side of the apparatus and in the *middle* of the outer zone. The animal was allowed to move freely for 10 min, and activities were video-tracked by an overhead camera. Heat maps were generated in the analysis of the video recording to reflect anxiety and explorative activities as illustrated in the *lower subpanel*, in that if the animal is more anxious then less time is spent in the inner zone. **(B, C)** Distance moved **(B)** and duration in the pre-defined inner zone **(C)** were collected in each 1 min period throughout the total 10 min test. *Solid lines* are the Young Group animals, and *dotted lines* are the Aged Group animals. The AUC was calculated for each age- and dose-defined group and depicted as the in-set figure. **(D)** Latency, or time taken to reach to the inner zone. *n* = 8–9/group in the Young Group and 8/group in the Aged Group. *Asterisk* indicates the significance of the difference *versus* the Control diet fed and age matched group; *p < 0.05 and **p < 0.01. All data were presented as mean ± SEM. See also Supplementary Figure S3. OF, open field. Color images are available online.

Young (9–10 weeks of age) and Aged (72–74 weeks of age) mice were assessed for anxiety-like behavior and locomotion by using an open field (OF) test (Fig. 3A). The Young group moved faster than the Aged group as expected (Supplementary Fig. S3A), thus serving as an initial control and validation for detecting differences between groups. Distance moved/path length was measured in 1 min intervals throughout the total 10 min period (Fig. 3B). A two-way analysis of variance (ANOVA) analysis of area-under-the-curve (AUC) showed a significant effect of age (*p* < 0.0001) whereas no effect of BPA itself (*p* = 0.3622). Within the young group, a one-way ANOVA analysis of AUC of distance moved showed a significant (*p* = 0.0009) reduction in young animals under LD BPA exposure compared with the young control (CTL) group (Fig. 3B).

The effect of BPA on anxiety-like behavior was assessed by measuring duration (Fig. 3C), latency (Fig. 3D), and frequency (Supplementary Fig. S3B) to the inner zone in the OF test. Generally, anxious animals show thigmotactic responses (56) (Fig. 3A). Aged animals explored the inner zone less, as revealed by a two-way ANOVA analysis of AUC for duration in the inner zone showing a significant effect of age (*p* < 0.0001) but not BPA (*p* = 0.5982) nor BPA × Age (*p* = 0.6136) (Fig. 3C). Interestingly, young animals under LD and HD BPA exposure tended to have increased latency to the inner zone (Fig. 3D). The increase in latency reached significance in aged mice that were peripubertally exposed to LD BPA (Fig. 3D). Two-way ANOVA of AUC of latency to the inner zone showed the significant effect of age (*p* = 0.0032), BPA (*p* = 0.0095) as well as BPA × Age interaction (*p* = 0.0161). Animals did not differ in the frequency to the inner zone (Supplementary Fig. S3B). As expected (50), a strong correlation between time in the inner zone and fecal counts was observed (Supplementary Fig. S3C). Taken together, peripubertal LD BPA produced immediate and long-lasting anxiogenic responses.

### Peripubertal HD BPA exposure impairs exploratory and spatial memory abilities

**FIG. 4.**
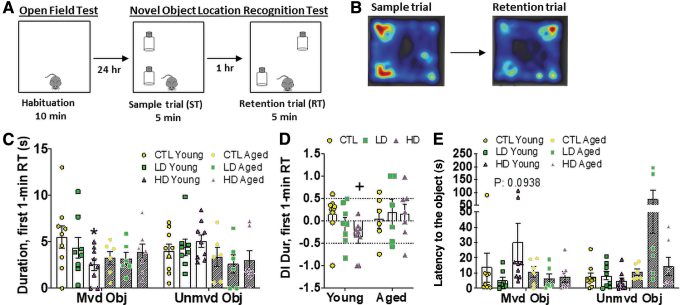
**Peripubertal HD BPA exposure impairs exploration and spatial memory as determined by NLR test. (A)** Experimental design of the NLR test. Testing occurred in an OF arena, to which the animals were first habituated in the OF test 1 day earlier. Two identical objects were introduced to the arena. The animal was allowed to explore the arena with the two objects for 5 min referred to as ST. Five minutes thereafter, the animal again encountered the two objects, except that one of them had a changed/novel location, referred to as RT. Both trials were video-tracked and scored to evaluate spatial memory function. **(B)** Illustrative heat maps generated during the NLR test. Generally, the animal spends equal time on two identical objects during the ST (*left subpanel*) and prefers to spend more time investigating the moved object in the novel location during the RT (*right subpanel*). **(C, D)** Duration at the moved (Mvd) or unmoved (Unmvd) object (ObJ) in the first minute of RT **(C)** and resultant discrimination index **(D)**. **(E)** Latency to the object in the RT. Clear bars are the Young Group, and shadowed bars are the Aged Group. *n* = 8–9/group in the Young Group and 6–7/group in the Aged Group. *Asterisk* indicates the significance derived from paired *t-*test of performance in the moved object *versus* unmoved object within the BPA dose-defined group; *p < 0.05. Plus indicates the significance derived from one-way ANOVA of the difference *versus* the Control diet-fed and age-matched group; ^+^*p* < 0.05. All data were presented as mean ± SEM. See also Supplementary Figure S4. ANOVA, analysis of variance; NLR, novel object location recognition task; RT, retention trial; ST, sample trial. Color images are available online.

The effect of BPA on spatial memory was assessed in the novel location recognition (NLR) (Fig. 4A). Generally, subjects spend equal time with two identical objects during the sample trial (ST) and then prefer to spend more time investigating the object moved to a novel location during the retention trial (RT), as shown in the representative heat maps (Fig. 4A, B). In scoring the total 5 min spent exploring both objects, paired *t-*tests within each age and dose group did not show a significant effect of BPA on place discrimination in both ST (Supplementary Fig. S4A) and RT (Supplementary Fig. S4B).

However, it is important to note that in this NLR test, there are no stimuli or rewards. Under these circumstances, mice lose interest as they start habituating to the environment, which was reflected by the decrease in locomotion in the OF task (Fig. 3B). Thus, the first minute bin of the 5-min RT was used for further analysis.

In the first minute of the RT, paired *t-*tests showed that HD BPA-, but not CTL- and LD BPA-groups, explored the moved object less relative to the moved object (*p* = 0.0237) (Fig. 4C). Importantly, a one-way ANOVA further showed a significant decrease in the discrimination index (DI) in the HD BPA group compared with CTL and the LD BPA group, which indicates spatial memory decline (*p* = 0.0376) (Fig. 4D). Consistently, paired *t-*tests in each group showed that HD BPA-exposed young males tended to increase latency to the moved object (*p* = 0.0938) (Fig. 4E). These effects were not observed in aged animals (Fig. 4C–E). Taken together, peripubertal HD BPA exposure impaired object location memory in young mice.

### Peripubertal HD BPA exposure limits performance in Y-maze spatial learning and memory tasks

**FIG. 5.**
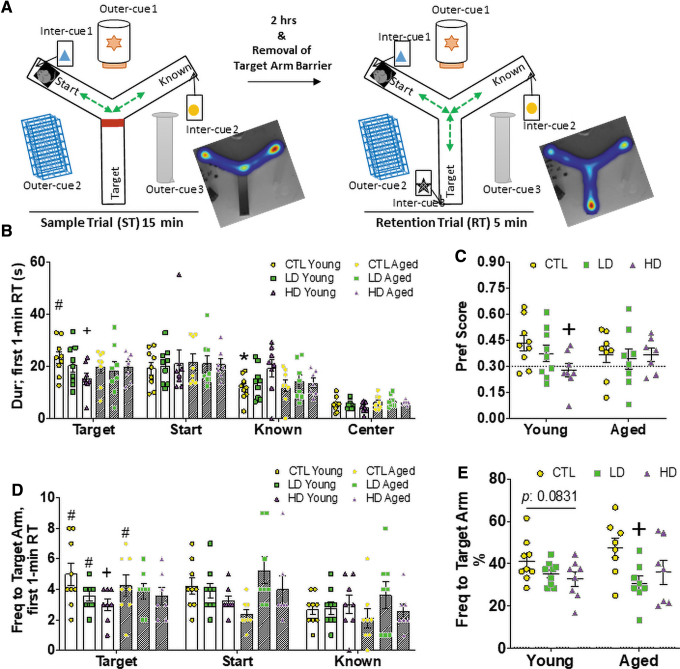
**Peripubertal HD BPA exposure limits performance in Y-maze spatial learning and memory tasks in young and aged mice. (A)** Experimental design of the Y-maze test. The Y-maze apparatus contains a Y-shaped maze with three *white*, *opaque* plastic arms. Cues inside and outside of the maze guide the animals for spatial learning. The target arm was blocked in the first trial referred to as ST and became accessible 2 h later in the RT. The animal was introduced in the start arm facing away from the center of the maze, and this allowed to explore the two unblocked arms as indicated in each trial. As illustrated in the heat maps, rodents generally explore equally in the accessible start and known arms during the ST, and they prefer to investigate the new released target arm during the RT rather than returning to the two that were previously visited. **(B, C)** Duration in each arm and the center in the first minute of RT **(B)** and resultant preference score **(C)**. **(D, E)** Frequency to each arm in the first minute of RT **(D)** and resultant percent frequency to the target arm **(E)**. Clear bars are the Young Group, and shadowed bars are the Aged Group. *n* = 8–9/group in the Young Group and 7–8/group in the Aged Group. *Asterisk* indicates the significance derived from paired *t-*test of performance in the known arm *versus* start arm; *p < 0.05. *Number sign* indicates the significance derived from paired *t-*test of performance in target arm *versus* known arm; ^#^*p* < 0.05. *Plus* indicates the significance derived from one-way ANOVA of the difference *versus* the Control diet-fed and age-matched group; ^+^*p* < 0.05. All data were presented as mean ± SEM. See also Supplementary Figure S5. Freq, frequency. Color images are available online.

We continued to assess the impact of peripubertal exposure of BPA on spatial learning and memory by using the Y-maze forced alternation test (Fig. 5A). Generally, rodents investigate a new arm of the maze (target arm) during the RT rather than returning to the two that were accessible (*i.e*., start arm and the second arm) during the ST (Fig. 5A).

For duration in each arm and the center during the ST, paired *t-*tests in each group showed that the CTL and BPA-exposed males spent comparable time in both arms (Supplementary Fig. S5A). Examining the entire 5-min scoring of the RT, young males of the CTL group, but not LD BPA or HD BPA, spent more time in the new released/novel target arm over the known arm (*p* = 0.0022) (Supplementary Fig. S5B), indicating that the BPA-exposed Young groups did not explore the novel environment. When the animals aged, the CTL group no longer exhibited preference to the target arm, leading to a comparable target arm investigation with the Aged BPA groups (Supplementary Fig. S5B), thus suggesting that aging has a more powerful influence on spatial learning and memory than BPA, and/or peripubertal BPA exposure no longer impacts spatial learning and memory at later ages.

Similar to NLR, the first minute bin of behavioral activities was extracted and scored. The arm preference in the young CTL group was consistently observed in the first minute (Fig. 5B). Importantly, a one-way ANOVA analysis in duration showed that HD BPA-exposed young mice spent less time in the target arm (*p* = 0.0414) (Fig. 5B), confirming the impaired explorative behavior. A one-way ANOVA analysis on preference score of duration showed impaired spatial memory further in HD BPA-exposed young males (Fig. 5C). Neither of the differences extended into later life (Fig. 5B, C).

For frequency to each arm and the center, paired *t-*test in each group showed that only young CTL- and LD BPA-exposed, but not HD BPA-exposed, had more visits to the target arm over the known arm (*p* = 0.0138 and 0.0070, respectively) in the total 5-min RT (Supplementary Fig. S5C). Similarly, arm preference was further observed when focusing on the first minute of the trial (*p* = 0.0081 and 0.0431, respectively) (Fig. 5D). Importantly, one-way ANOVA analysis showed that the HD BPA-exposed Young Group had less visits to the target arm when compared with the CTL group (Fig. 5D, E) and reflects a spatial memory deficit in the HD BPA group, similar to what was observed for the DI in the novel location recognition test (Fig. 4D). In addition, the HD BPA-exposed Young Group showed a slight increase in latency to the target arm during the RT (Supplementary Fig. S5D).

In regards to the Aged Group, BPA-exposed animals, but not Controls, failed to show explorative interest over the start arm in the analysis of either frequency to target arm (Fig. 5D) or percent frequency to target arm (Fig. 5E). Taken together, the Y-maze test suggests that peripubertal HD BPA exposure impaired spatial memory in young mice, with this deficit somewhat lingering into later life.

### Peripubertal HD BPA exposure attenuates tissue-specific H_2_S production capacity and elevates renal oxidative stress

Previous studies on the neurological effects of BPA focused on impaired endocrine signaling, but the molecular mechanisms remain unclear. Recently, BPA exposure in adult rats was shown to reduce reactive oxygen species (ROS) scavenging capacity and induce systemic oxidative stress (33). Meanwhile, endogenously produced H_2_S is a redox-modifying bioactive gasotransmitter with numerous roles in metabolism (70), prevention of aging-related neurodegeneration/cognitive decline (19, 48), and endocrine homeostasis (23, 58). The enzymes that produce H_2_S in mammals are partially regulated by estrogen (75), TH, and growth hormone (23). Therefore, we hypothesized that peripubertal exposure to BPA increases oxidative stress concurrent with diminished anti-oxidant H_2_S production capacity and/or downstream persulfidation signaling, which would ultimately contribute to the cognitive decline and memory deficits observed in the present study.

**FIG. 6.**
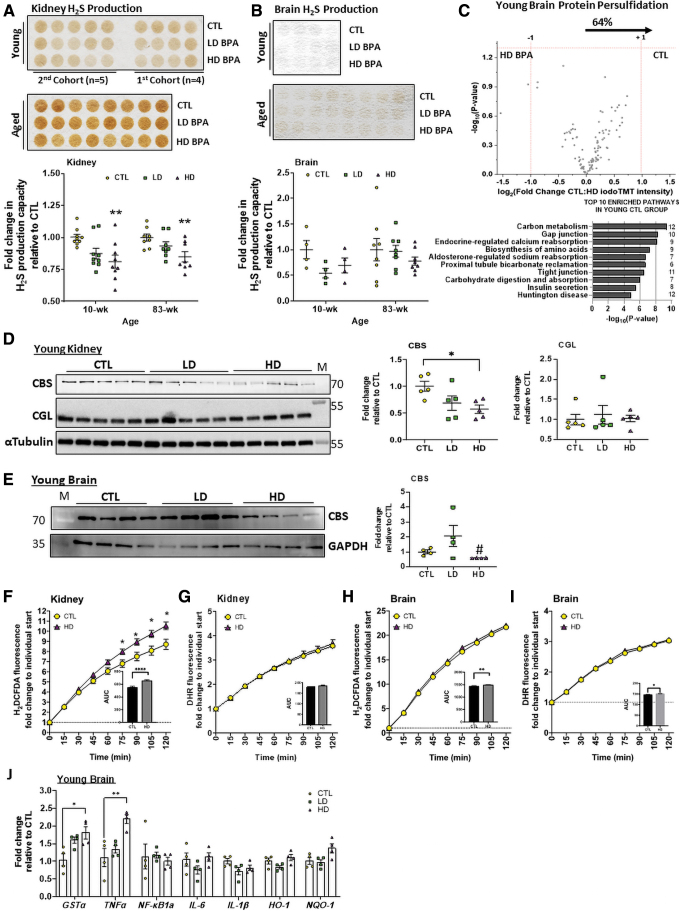
**HD peripubertal BPA exposure attenuates H_2_S production capacity and/or persulfidation. (A, B)** H_2_S production capacity measurement *via* the lead acetate/lead sulfide assay in kidney **(A)** and brain **(B)** of Young and Aged Group animals. *n* = 9/group in Young Group for kidney whereas 4/group in brain samples. *n* = 8/group in Aged Group for kidney and brain. *Asterisk* indicates the significance of the difference *versus* the Control diet-fed and age-matched group; **p < 0.01, determined by one-way ANOVA with Dunnett's multiple-comparison test. **(C)** *Upper panel*: Volcano plot showing differentially abundant persulfidated proteins in brain from CTL (*n* = 3/group) and HD BPA (HD, *n* = 3/group) exposed Young Group mice as detected *via* the biotin thiol assay and subsequent iodoTMT labeled mass spec identification and quantification of peptides. The Log_2_(Fold change CTL:HD) *X*-axis displays average fold change in iodoTMT intensity for each identified persulfidated protein/peptide, whereas the −log_10_ *Y*-axis displays calculated *p* value when comparing intensity for each identified persulfidated protein from CTL *versus* HD BPA-exposed mice with *p* value obtained from the two-tailed Student's *t-*test. The percentage and direction of proteins skewed toward CTL is provided. *Lower panel*: KEGG biological pathway enrichment *via* g:Profiler of the persulfidated proteins skewed for enrichment in the CTL group. **(D, E)** Immunoblots of CGL and/or CBS in kidney **(D)** and brain **(E)** of Young Group mice. *n* = 5/group in assaying kidney samples whereas 4/group in assaying brain samples. Expression was normalized to either αTubulin or GAPDH. *Asterisk* and *pound* indicate the significance of the difference *versus* the Control diet-fed group; * or ^#^*p* < 0.05. Specifically, a Student *t-*test was performed for the brain samples between the HD BPA group and the CTL group. **(F–I)** Reactive oxidative and nitrosative stress in the kidney (**F, G**) and brain (**H, I**) of Young Group mice exposed to CTL diet and HD BPA diet as detected by the H_2_DCFDA and DHR123 probes. *n* = 9/group for kidney and 4/group for the brain. *Asterisk* indicates the significance of the difference *versus* the CTL group; *p < 0.05, **p < 0.01, and ****p < 0.0001. Specifically, a Student *t-*test was performed at each kinetic reading point between the HD BPA group and the CTL group. **(J)** qPCR analysis of redox and inflammatory genes in brains of Young Group mice, *n* = 4/group. *Asterisk* indicates the significance of the difference *versus* the Control-matched group; *p < 0.05 and **p < 0.01, with the statistical test being a one-way ANOVA with Dunnett's multiple-comparison test. All data were presented as mean ± SEM. See also Supplementary Figure S6. CBS, cystathionine β-synthase; CGL, cystathionine γ-lyase; CTL, control; DHR123, dihydrorhodamine 123; GAPDH, glyceraldehyde 3-phosphate dehydrogenase; H_2_DCFDA, 2′,7′-dichlorofluorescin diacetate; qPCR, quantitative polymerase chain reaction. Color images are available online.

We first examined the impact of BPA exposure on immediate and later-life H_2_S production capacity in two major H_2_S-producing organs: liver and kidney (71). Renal, but not hepatic, H_2_S production capacities in young and aged HD BPA groups were reduced relative to the CTL group (Fig. 6A and Supplementary Fig. S6A). We next examined H_2_S production capacity in hippocampus-containing brain tissues due to our findings on the hippocampal-dependent memory deficits (Figs. 4 and 5 and Supplementary Figs. S4 and S5). No significant differences were detected in young or aged brain samples, although there was a trend of reduced H_2_S production capacity in HD BPA-exposed groups (Fig. 6B).

A similar trend was observed when examining protein persulfidation (R-SS_n_H) in hippocampus-containing brain tissues of the Young Group mice. The HD BPA-exposed mice had decreased numbers and/or abundance of persulfidated proteins compared with CTL mice by 36% (Fig. 6C). Biological function and pathway enrichment *via* g:Profiler analysis (51) utilizing the KEGG database (30) of the persulfidated proteins skewed toward the Young CTL group revealed 43 pathways, with carbon metabolism, gap junction, and endocrine-regulated calcium reabsorption as the top 3 (Fig. 6C).

Consistent with the earlier mentioned shifts in H_2_S production capacity and signaling, immunoblots revealed decreased protein expression of CBS, but not cystathionine γ-lyase (CGL), in the kidney (Fig. 6D) and hippocampus-containing brain (Fig. 6E) from the HD BPA-exposed Young Group. Thus, peripubertal exposure to HD BPA leads to acute and/or long-lasting deficiencies in renal H_2_S production and brain persulfidation, along with suppression of renal and brain CBS protein expression.

Attenuation of H_2_S production and/or H_2_S-producing enzyme expression are associated with elevated oxidative stress in rodents (2, 31, 57) and humans (14, 61). Therefore, we next examined HD BPA-induced oxidative and/or nitrosative stress by using the probes 2′,7′-dichlorofluorescin diacetate (H_2_DCFDA), aka DCFHDA, and dihydrorhodamine 123 (DHR123) in the kidney (Fig. 6F, G) and brain (Fig. 6H, I). In renal lysates, the HD BPA group increased the conversion of H_2_DCFDA to the fluorescent product, 2′,7′-dichlorofluorescein (DCF), at kinetic reading periods from 75 min through 120 min, indicating elevated ROS (Fig. 6F). Consistently, a *t*-test analysis of AUC in fold-change fluorescence showed a significant increase in HD BPA-exposed mice. However, detection of reactive nitrogen oxide species by the DHR123 probe did not reveal differences between HD BPA and CTL renal lysates (Fig. 6G).

Similarly, in the analysis of BPA-induced oxidative stress in hippocampus-containing brain lysates, the HD BPA group did not have elevated fluorescence in either H_2_DCFDA or DHR123 assays at specific time points, but AUC *t*-test analysis exhibited slight increases in the HD BPA group for both probes (Fig. 6H, I). As the HD BPA group did not have strong induction of oxidation stress in the brain, we next examined the expression of genes involved in redox homeostasis and inflammation in the brain. The HD BPA treatment increased expression specifically of glutathione S-transferase (*GST*) α and tumor necrosis factor alpha (*TNF*α) (Fig. 6J), with no such differences seen in the other genes of interest (Fig. 6J). Thus, peripubertal exposure to HD BPA reduced H_2_S production capacity while elevating oxidative stress in the kidney, and elevated neuroinflammatory and cognitive impairing (21) *TNF*α expression in the brain.

### Multi-component correlative analysis reveals connections between H_2_S production, oxidative stress, and cognitive performance

Pearson correlation analysis was performed to determine the relationship between variables in this study, with a focus on renal and brain H_2_S production capacity and/or H_2_S-producing enzyme (CGL and CBS) expression correlating with memory deficits derived from BPA exposure. There was a positive correlation between renal H_2_S production capacity and frequency to the target arm in the Y-maze test when taking into account all three groups with a total sample size of 26 (*r* = 0.39, *p* = 0.050) (Supplementary Fig. S7A). The correlation was stronger when examining renal H_2_S production capacity with the percent frequency to the target arm normalized to total visits (*r* = 0.48, *p* = 0.013) (Supplementary Fig. S7A).

**FIG. 7.**
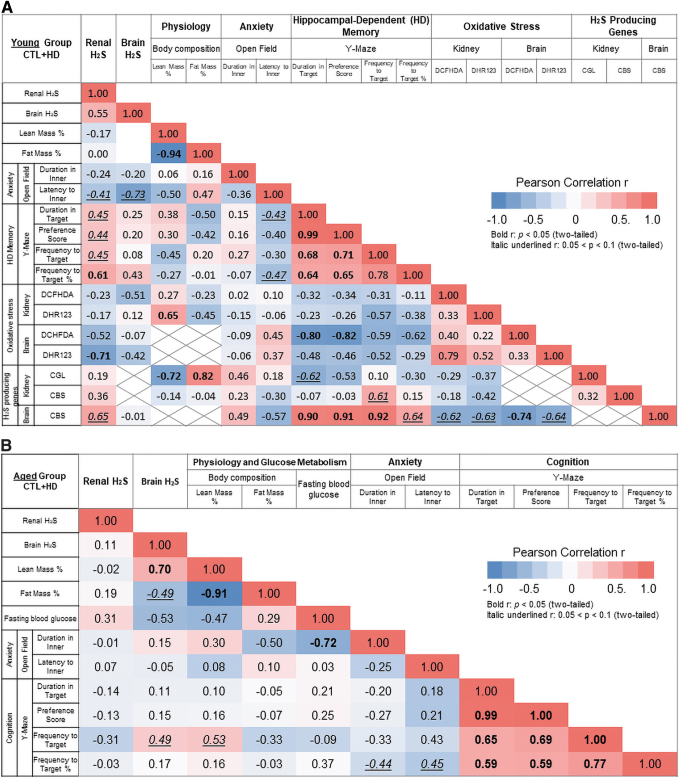
**HD peripubertal BPA exposure attenuated renal H_2_S production capacity and brain CBS expression, which correlated with BPA-induced memory deficits in young mice. (A, B)** Pearson correlation analysis between variables as indicated in the Young **(A)** and Aged **(B)** Group mice peripubertal fed with Control and HD BPA diets. *n* = 10–18/group in the Young Group and 15–16/group in the Aged Group. Pearson correlation coefficients also referred to as *r* values between two variables were annotated in the box. *Red color* indicates positive correlations and *blue color* indicates negative correlations, with their intensity being proportional to the *r* values. The *color legend* in the *right side* of the chart indicates the *r* values and the corresponding colors. *Bolded numbers* indicate reaching statistical significance with *p* < 0.05, whereas *underlined italic numbers* indicate approaching statistical significance with 0.05 < *p* < 0.1. See also Supplementary Figure S7. Color images are available online.

Notably, in analyzing specifically CTL and HD BPA-exposed groups with a total sample size of 17, positive correlations between renal H_2_S production capacity and all the Y-maze test variables were observed, including duration in the target arm (*r* = 0.45, *p* = 0.073), preference score (*r* = 0.44, *p* = 0.075), frequency to the target arm (*r* = 0.45, *p* = 0.072), and percent frequency to the target arm (*r* = 0.61, *p* = 0.010) (Fig. 7A). Conversely, the correlation analysis between brain H_2_S production capacity and hippocampal-dependent memory endpoints did not show a close relationship (Fig. 7A and Supplementary Fig. S7A). However, in analyzing specifically the CTL and HD BPA exposed groups (sample size = 8), brain CBS protein expression had a strong positive correlation with all Y-maze variables, including duration in the target arm (*r* = 0.90, *p* = 0.002), preference score (*r* = 0.91, *p* = 0.001), frequency to the target arm (*r* = 0.92, *p* = 0.001), and percent frequency to the target arm (*r* = 0.64, *p* = 0.088) (Fig. 7A).

Interestingly, renal H_2_S production capacity negatively correlated with brain DHR123-detected oxidative/nitrosative stress (*r* = −0.71, *p* = 0.046) (Fig. 7A). The aged group did not show a significant correlation between renal and brain H_2_S production capacity with other variables (Fig. 7B and Supplementary Fig. S7B). Taken together, decreased renal H_2_S production capacity and brain CBS protein expression negatively correlated with BPA-induced memory deficits in young mice; meanwhile, brain CBS protein expression negatively correlated with BPA-induced ROS accumulation in young mice.

## Discussion

**FIG. 8.**
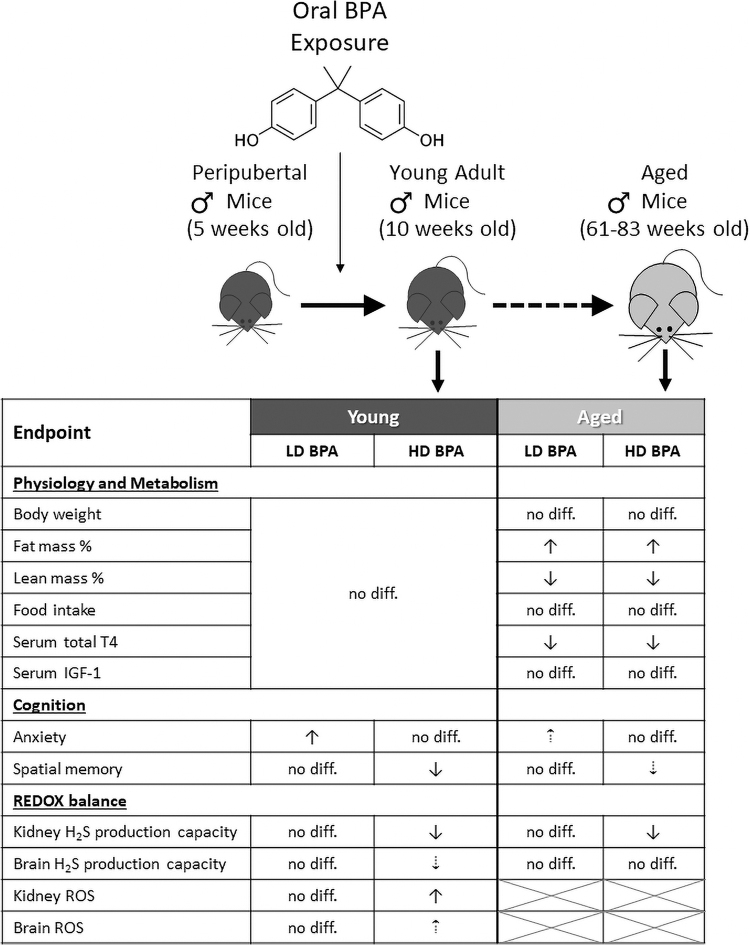
**Model of peripubertal BPA-induced alterations in physiology, metabolism, and cognitive development.** Male C57BL/6J mice at 5 weeks of age were orally exposed for 5 weeks to 250 μg BPA/kg per day defined as LD group (LD BPA), or 250 mg BPA/kg per day defined as HD group (HD BPA). Both LD and HD BPA exposure did not have an acute impact on BM composition, serum IGF-1, or serum tT4, but they did decrease LM and increase FM without affecting total BM as the animals aged. These changes were accompanied by decreased serum total TH and impaired glucose metabolism that was independent of food intake. In addition, LD BPA had an anxiogenic effect whereas HD BPA caused cognitive and behavioral deficits in young mice, with these deficiencies somewhat lingering into late life. Notably, HD BPA attenuated brain CBS expression, which correlated with spatial memory deficits as well as BPA-induced ROS accumulation in young mice. Meanwhile, HD BPA decreased renal H_2_S production capacity acutely and long-term, which correlated with BPA-induced memory deficits in young mice. These findings provide molecular insights for BPA-induced biological insults, which suggests that peripubertal BPA exposure diminishes antioxidant renal H_2_S production capacity, thereby possibly impacting the progression of aging and aging-associated diseases. Our results bolster the need for improved monitoring and limitation of adolescent BPA exposure. *Large solid arrows* indicate differences detected that reached statistical significance, whereas *smaller dashed arrows* indicate differences detected that were trending. IGF-1, insulin like growth factor 1; TH, thyroid hormone; ROS, reactive oxygen species.

In our current study, we examined the immediate and lifelong effect of peripubertal BPA exposure on physiology, metabolism, and neurobehavioral outcomes in male C57BL/6J mice. The data showed that peripubertal BPA exposure impacted long-term body composition by increasing FM and decreasing LM. The LD/environmentally relevant (55) 250 μg/day/kg b.w. BPA exposure had anxiogenic effects, whereas HD 250 mg/day/kg b.w. BPA caused cognitive and behavioral deficits by decreasing spatial memory function primarily in young mice that somewhat persisted in aged mice. Notably, we revealed that HD BPA weakens renal H_2_S production capacity acutely and in a life-long manner, which further correlated with spatial memory deficits in young animals. These results are summarized in Figure 8.

In the following section, we discuss our findings in the context of previous species- and strain-specific sensitivities to different BPA dosages due to differences in timing and route of administration, diet background, and modeling. We also describe implications of disrupting ER-regulated locomotor activity and anxiety *via* peripubertal BPA exposure, and the link between BPA and H_2_S-related outcomes in metabolism, behavior, and aging. Ultimately, we discuss how our study may impact personal and regulatory agency policies on BPA usage.

Species- and/or strain-specific sensitivities to BPA exposure most likely account for inconsistent and sometimes contradictory results in non-human BPA studies. Thus, their selections should be responsive to relevant environmental, epidemiological, and biomedical trends in BPA contamination and routes of exposure (40). Likewise, doses of BPA not found to be toxic in traditional toxicological studies conducted for risk assessment and thus not considered on the toxicity scale may still have aversive capabilities [U.S. Environmental Protection Agency Integrated Risk Information System (64a)]. In the initial 1982 National Toxicology Program (NTP), B6C3F1 mice showed insensitivity to a 50 mg/day/kg b.w. and this dose was accepted by the U.S. Food and Drug Administration (U.S. FDA) as the LOAEL; however, this same dose drove body weight reduction in F344 rats (43).

Strain-specific sensitivity, in addition to species-specific sensitivity to BPA, has also been reported (55), highlighted by the insensitivity of Sprague-Dawley rats to BPA, especially in reproductive endpoints, compared with other rat stains such as F344 rats and Wistar rats. For example, exposing F344 rats for 3 days with ∼300 μg/kg/day of BPA resulted in hypertrophy, hyperplasia, and mucus secretion in the uterus and hyperplasia and cornification of the vaginal epithelium, all of which were not observed in Sprague-Dawley rats following the same BPA treatment (60). Sprague-Dawley rats were also revealed to be resistant to BPA doses up to 300 mg/kg/day in uterotrophic bioassays, despite this strain showing a significant response to ethinyl estradiol at just 0.3 μg/kg/day (25). Thus, it is not surprising that the recent BPA risk assessment performed from the CLARITY-BPA program in Sprague-Dawley rats exposed pre- and/or postnatally to various BPA doses between 0 and 25 mg/kg/day b.w. concluded that BPA use is relatively safe (44).

Potential underlying reasons as to strain-specific differences in BPA sensitivity may rest in inherent sensitivities to endogenous endocrine signals due to many laboratory rodents selected for rapid postnatal growth. Besides the marked difference in strain/species sensitivity, epigenetic modulation could also account for the different results observed. The BPA can affect DNA methylation regulator genes that possibly underlie its sexually dimorphic phenotypes in brain function and behavior. In light of these points, along with recent and remarkable studies showing human BPA exposure has been dangerously underestimated (17) and is significantly associated with increased risk of all-cause mortality (4), we felt it warranted in this study to revisit the health effects of BPA exposure and address the unknowns regarding the developmental timing of BPA exposure in immediate and age-associated endpoints utilizing a wide dose range in another species/strain of rodent that is sensitive to external toxicological and pharmacological insults.

The peripubertal period is increasingly recognized as crucial for growth and development. During this period, the growth rate in humans is comparable or even exceeds the rate at age two (63a). It is also recognized as a crucial time for maturation of multiple organ systems, major changes in the central nervous system, and cognitive development (9). Although disturbance metabolic programming from BPA is recognized (1, 3, 16, 37, 54, 72), studies specifically examining the acute and long-term effects of peripubertal BPA exposure are limited and results are confounded by factors such as, in addition to the species/strain issues highlighted earlier, the route of exposure or diet composition.

A study of CD-1 mice (54) compared two exposure windows: “perinatal (GD 8–PND 21)” *versus* “perinatal + peripubertal (GD 8–PND 35),” of BPA at 0.25 μg to 250 μg/kg b.w./day *via* osmotic minipumps and then examined until 34 weeks of age. The BPA exposure at the early perinatal window showed an overall elevation in body weight, with the dose of 25 μg BPA further causing an increase in adiposity and a decline in percent LM. Interestingly, extending the window of BPA exposure through the peripubertal period notably reduced the differences between exposure groups in male mice. It should be noted that exposure routes such as the osmotic minipumps mentioned earlier may not fully recapitulate normal human exposure. The BPA exposure to humans is mainly through the daily use of beverage bottles, food cans, food containers, thermal receipts, and baby bottles (36). Thus, diet- or drinking water-based exposure routes, such as the ones used in our current study, are considered to be more reflective of actual BPA exposure in humans.

In another study using C57BL/6J mice (72), a similar window of exposure as to what we used in the current study with the same mouse strain (beginning at 5 weeks of age for 30 days *vs.* 35 days in this study) was utilized with BPA (5 μg to 5000 μg/kg b.w./day) incorporated into low-fat diets (10% kcal in fat), which resulted in acute body weight and FM increases in a non-monotonic dose-dependent manner in both genders. However, no differences were found when the BPA was incorporated into a higher fat diet (35% kcal in fat) (72), highlighting how diet background is a confounding variable in BPA exposure studies. Here, we used a BPA-infused diet containing 15.8% kcal in fat, which did not cause acute changes in body weight or body composition during the exposure period, but did alter body composition in the long-term during aging. Their observations (72) combined with our present study lend credence that peripubertal BPA exposure impacts body weight and body composition.

However, the extent and possible mechanisms involved are likely dependent on the background diet composition and the window of time during which these factors are examined. Although we did not find peripubertal BPA exposure immediately impacting circulating TH levels nor food intake, suggestive that the doses were not too severe, we ultimately found that decreased T4 levels in the aged TH activity have strong roles in both fat and glucose metabolism (41), and we suspect that it could be these latent changes in T4 levels that led to BM composition alterations.

The lipophilic structure of BPA enables itself to easily travel across the blood–brain barrier (62) and directly affect the central nervous system, permitting its anxiogenic effect (46) and the impairment in cognition development (6, 13). However, studies examining long-term effects of BPA on anxiety and cognition during aging are limited. Both maternal BPA exposure in C57BL/6J mice (11) and gestational–lactational exposure in deer mice (*Peromyscus maniculatus*) (59) showed an anxiogenic effect, but it was only tested in later juvenile stages (PND 26–30) and young adult stages (not specified, but estimated at PND 80). In another study using Wistar rats, the anxiogenic effect of birth-to-puberty BPA exposure was only observed in the juvenile/prepubertal stage (PND 24–28) and did not strongly persist into young adulthood (PND 60–70) (47).

Similarly, in our current study, we showed the acute anxiogenic effect of peripubertal LD BPA exposure as evidenced with decreased locomotor activity and increased anxiety in the OF test, which only weakly persisted during aging in mice 18 months of age. When examining cognition, in the CLARITY-BPA program, they found that Sprague-Dawley female but not male rats exposed to prenatal–postnatal 2500 μg BPA/kg b.w. exhibited impaired spatial learning in the Barnes maze test when tested at young adulthood (PND 90) (29). Male deer mice with their dams fed with 50 mg BPA/kg diet throughout mating, gestation, and lactation also showed impaired spatial learning in young adult/adolescent ages (PND 60) (59).

Moving onto previous studies that examined rodents at later ages, CD-1 mice exposed to 100 mg/L BPA through drinking water (with estimated 25 mg/kg b.w. daily intake) during PND 28–56 resulted in decreased spatial memory at 18 months of age when tested in the novel object location recognition and water maze assessments (28). In our study, HD BPA of 250 mg/day/kg b.w. during peripubertal exposure decreased spatial memory function primarily in young mice that somewhat persisted into aged mice.

Long-term effects of early life BPA exposure on physiological functions in C57BL/6J male mice were examined in this study. We showed that the weight-gain (54) and TH-related endocrine disrupting (53) properties of BPA exposure become more prominent in late life, causing decreased TH, impaired glucose tolerance, and increased FM. This typically mimics the presentation of hypothyroidism in humans (34). However, we did not see alterations in food intake in the BPA-exposed mice, suggesting that the changes in body composition and the impaired glucose metabolism are not due to caloric intake, but rather changes in metabolic programming related to glucose and fat utilization and storage. As BPA acts as a weak ER agonist (12) and TR antagonist (41) along with the disruptor of renal H_2_S production and brain persulfidation as shown here, it could mechanistically interfere with metabolic and cognitive programs *via* these aberrations as all these factors play fundamental roles in metabolism and behavior (38, 39, 70).

Importantly, our finding of acute and persistent decreases in renal H_2_S production capacity could pose as an unexpected causative mechanism for the physiological, metabolic, and cognitive impact of BPA treatment. Suppressed H_2_S levels or deficiencies in the enzymes that produce it have been similarly linked to physiological (68), metabolic (23), and cognitive (48) declines. The interface of H_2_S and TH activity has been previously reported in the liver, whereas TRβ when coupled to TH is a suppressor of CGL expression and subsequent H_2_S production (23). However, our findings here suggest that this interface may be tissue specific, with systemically decreased TH signaling resulting in reduced renal H_2_S production.

Alternatively, it is important to note that the prior publication (22) showed that TRβ can act as a suppressor as well as a stimulator of CBS and CGL expression and/or H_2_S production in the liver or liver-derived cells, with this dichotomy based on its ability to bind T3 (or T4). Thus, although BPA may interfere with T3 (or T4) interacting with TRs, it still can stimulate and/or inhibit TR-mediated downstream gene transcription, which can be largely dependent on the TR subtype present in specific tissues (40). As the half-life of BPA in the body in only ∼5 h, this suggests multiple mechanisms, potentially epigenetic in nature, giving rise to reduced renal H_2_S production at both young and aged time points. Although these acute and long-term mechanism(s) underlying BPA-induced inhibition of renal H_2_S production and its relationship with the observed hypothyroid phenotype are not clear currently, our study provides insight into BPA-induced physiologic, metabolic, and cognitive declines and reveals the possible involvement of attenuated renal H_2_S.

In addition, we showed that HD BPA-induced spatial memory deficits in young animals correlated with the HD BPA-induced reduction of renal H_2_S production and brain CBS expression. This points to a potential link between BPA and renal H_2_S- and redox-related outcomes in memory formation. Although still in its infancy, reduced kidney H_2_S production and/or H_2_S-producing protein expression are revealed to be associated with elevated oxidative stress in rodents (2, 31, 57) and humans (14, 61). Oxidative stress further promotes cellular susceptibility and ultimately contributes to kidney pathogenesis and disease development (14). Kidney dysfunction from either acute kidney injury (35) or chronic kidney disease (27) contributes to dysfunction of other distant organs in patients. One of the most often affected organs is the brain, leading to both peripheral and central neurological disorders, such as cognition decline (27, 35). Our finding that HD BPA increased neuroinflammatory *TNF*α expression, which has been shown to impair memory (21), suggests renal oxidative stress and H_2_S decline combined with brain inflammatory signaling contribute to cognitive decline.

## Innovation and Conclusion

In summary, we found that peripubertal oral BPA exposure negatively impacts metabolic programming and emotional/cognitive behaviors primarily in young mice that can persist in aged mice. Notably, we revealed that BPA weakened renal H_2_S production capacity correlated with spatial memory deficits in young animals, which may help elucidate the mechanism of action for which BPA acts. Further, BPA induced lifelong suppression of renal H_2_S production capacity, highlighting its role in the increased risk of all-cause mortality with aging (4). Our study ultimately provides insights into the acute and latent molecular and physiological changes induced by environmental EDCs and gives evidence to support further research into limiting their exposure during peripubertal development (see Graphical Abstract).

Our study proposes for the first time that BPA exposure during the specific peripubertal period induces attenuation of renal and brain H_2_S production and/or persulfide signaling concomitant with increased ROS and/or reactive nitrogen species (RNS) stress, thereby possibly impacting the progression of aging-associated disorders. In terms of innovation, our results can be used to model and predict the potential long-term dangers unknowingly imposed on children as they grow into adulthood and later life. Likewise, the results can be used as a conceptual tool to develop targeted interventions to address the BPA-triggered health issues.

## Materials and Methods

### Animal studies and BPA exposure

All experiments were performed with the approval of the Institutional Animal Care and Use Committee (IACUC) from the Cleveland Clinic Lerner Research Institute, protocol number 2016-1778. Animals were male C57BL/6 (Jackson Laboratory) and maintained under standard housing conditions in the Cleveland Clinic Biological Resource Unit with *ad libitum* access to food and water, 12-h light/12-h dark cycles, and temperature between 20°C and 23°C with 30–70% relative humidity.

Two cohorts of mice were used in the study. The first cohort of animals was bought from the Jackson Laboratory (Stock No. 000664), shipped at 3 weeks of age, and acclimated at the Cleveland Clinic Biological Resources Unit for 1 week. The second cohort of animals was from C57BL/6 breeders originally obtained from Jackson Laboratory (Stock No. 000664). Mice were randomly assigned to experimental cages. Both cohorts of animals were given a customized diet (Research Diet No. D10012G) containing no added BPA for 3 days for the purpose of food acclimation and assessing for food intake. The animals were then further randomly assigned to one of three diets at 5 weeks of age and group housed: (i) Control diet containing no added BPA with *n* = 17 total mice, (ii) 250 μg BPA/kg b.w. per day defined as the LD BPA group with *n* = 17 total mice, and (iii) 250 mg BPA/kg b.w. per day as the HD BPA group with *n* = 17 total mice. The BPA was added directly to the customized research diet formulation or oral administration, with the dose calculated on the empirically determined per cage food intake and average animal mass per cage. The mice were maintained on these diets *ad libitum* for 5 weeks. One third of the animals of the first cohort and the whole second cohort were used as the Young Group, and tissues were sampled at 10 weeks of age. Two thirds of the animals of the first cohort referred to as the Aged Group were placed back on the standard rodent chow control diet (ENVIGO No. 2918) from the Cleveland Clinic Biological Resource Unit after 5 weeks of BPA experimental diet exposure and kept on this diet throughout all subsequent experiments. The compositional information for this diet is as follows: 18.6% protein, 44.2% carbohydrate, and 6.2% fat. Mice were euthanized *via* isoflurane exposure and cervical dislocation with subsequent tissue sampling at 83 weeks of age. The number of individual animals in each group analyzed per test or assay is provided in the respective figure legends.

### Metabolic parameters

Body composition was determined with an EchoMRI body composition analyzer and EchoMRI mouse immobilizing tubes (EchoMRI, Houston, TX) without the need for anesthesia. Blood glucose tolerance test (GTT) was performed in animals after a 4-h fast by intraperitoneal injection of 20% glucose solution in a volume (μL) equivalent to the value of 10 times of body weight (g). Blood glucose was measured with a handheld Contour^®^ Blood Glucose Monitor (Ascensia Diabetes Care US, Inc., Parsippany, NJ) from blood sampled from the tail vein. Serum IGF-1 and tT4 were measured with enzyme-linked immunosorbent assay (ELISA) kits (IGF-1, Cat. No. MG100, R&D System; tT4, Cat. No. KA0200, Abnova) following the manufacturer's manuals.

### OF test and novel location recognition test

All behavioral tests, including OF test, novel location recognition test, and Y-maze test, were performed for both Young and Aged Group animals in a continuous manner at least 48 h apart and conducted during the light phase of the daily cycle during the same period of the day in the Cleveland Clinic Rodent Behavior Core. The animals were first handled on 3 consecutive days (2 min per mouse on the first day, 1 min each on the second+ days) by the experimenter before the behavioral test to diminish experiment-induced anxiety. On the day of each behavioral test, the animals were transferred to the behavioral room and left undisturbed for 1 h before and after the test. The animals were tested in a random order. The test was then started in a dimmed environment. After each animal, the fecal bolus was counted and removed. The test apparatus was thoroughly wiped with 70% ethanol. The behavioral activities were video-traced by an overhead camera and analyzed/scored in EthoVision XT 13.0 software (Noldus, Wageningen, the Netherlands).

The OF apparatus was a square open box (40.5 cm H × 61.0 W × 51.0 D) and virtually divided into two arenas: outer and inner. Both the outer and inner areas shared the same center point, and the inner arena was set at 0.5 ratio of the outer arena area size. Each animal was released from the same side of the apparatus and in the middle of the outer zone. The animal was allowed to move freely for 10 min. The activity was collected in 1-min intervals and summed over the entire 10-min testing session (total activity). Behaviors assessed included total distance traveled (mm), velocity (mm/s), duration in the inner area (s), latency to the inner zone (s), and frequency to the inner zone.

The novel location recognition test occurred 24 h after the OF test in the same OF arena that the animals were habituated to. Therefore, there was no additional handling for the test. Two identical objects were introduced to the arena. Each animal was allowed to explore the arena with the two objects for 5 min in the trial referred to as ST. Five minutes thereafter, in the trial referred to as the RT, the animal again encountered the two objects, except that one of them had a changed/novel location. Behavior assessed included velocity (mm/s), investigation duration in the unmoved object and the moved object (s), latency to investigate the moved object (s), and frequency to the moved object. A DI was further calculated in the following equation to indicate the distribution of time in both objects:

$$DI=\frac{DurinMovedObject\left(s\right)-DurinUnmovedObject\left(s\right)}{DurinMovedObject\left(s\right)+DurinUnmovedObject\left(s\right)}$$

### Y-maze test

The Y-maze apparatus contained a Y-shaped maze with three arms (38.5 cm H × 9 cm W × 12 cm D) at a 120° angle from each other. Visual cues inside and outside of the maze assisted the mice with spatial familiarity. The task consisted of two trials with a 2 h interval. In the first trial called ST, the target arm was blocked with a barrier. The animal was introduced in the start arm facing away from the center of the maze, and this allowed to explore the two unblocked arms for 15 min. At the end of the ST, the animal was returned to its home cage in the behavioral testing suite. After 2 h, in the second trial called RT, all arms were accessible and the animal was introduced into the same start arm as in the ST and it was allowed to explore all three arms of the Y maze for 5 min. The behavior assessed included duration in the different arm (s), and frequency or the number of entries to the arm, as well as latency to the new released target arm (s). A preference score was calculated with the following equation to indicate the distribution of time spent in the target arm:

$$Preferencescore=\frac{DurinTargetArm\left(s\right)}{DurinStartArm\left(s\right)+DurinKnownArm\left(s\right)+DurinTargetArm\left(s\right)}$$

### Collection of frozen hippocampus-containing brain sections

On the day of tissue sampling, the brain was quickly removed from euthanized mice, flash-frozen in liquid nitrogen, and transferred to −80°C for storage. Brain sections ∼2 mm in thickness were collected according to the instructions from Wager-Miller *et al*. (66). Brain sections with hippocampal regions were used for H_2_S production capacity, persulfidation assays, and oxidative stress measurements.

### H_2_S measurements

H_2_S production capacity was measured by using the lead acetate/lead sulfide method (5, 24). Briefly, brain, liver, or kidney tissue was first placed in 1.5 mL microcentrifuge tubes containing 250 μL of 1 × passive lysis buffer (Promega) and homogenized, followed by multiple rounds of flash freezing/thawing by using liquid nitrogen. After homogenization and lysis, protein concentration was measured with bicinchoninic acid assay (BCA) kit (Bio-Rad) followed by normalization of proteins *via* additional 1 × passive lysis buffer. Next, the lead acetate/lead sulfide assay was set up by initially preparing the reaction mixture of 10 m*M* l-cysteine (No. 168149; Sigma) and 1 m*M* pyridoxal phosphate (PLP; No. 9255; Sigma) in phosphate buffered saline (PBS), with 150 μL placed into each well of a 96-well plate. One hundred micrograms of protein from each tissue or 20 μL of plasma was added to each respective well. Then, the plate was overlaid with lead acetate embedded filter paper and incubated at 37°C until lead sulfide was detected for quantification by using ImageJ densitometry analysis *via* the IntDen function after subtracting background levels obtained from reaction mixture-only wells with no tissue or protein added.

### Cellular oxidative stress detection with chemical probes H_2_DCFDA and DHR123

Kidney and brain sections were homogenized in Pierce^™^ RIPA lysis buffer (No. 89901; Thermo) with the addition of Halt^™^ Proteinase inhibitor cocktail (No. 78429; Thermo) for whole tissue protein extraction. Cellular oxidative stress was measured by using the probe-based microplate reader method. Briefly, 150 μg tissue homogenate was added to the 150 μL PBS containing 50 μ*M* of probe, either H_2_DCFDA (No. D6883; Sigma) or DHR123 (No. D23806; Invitrogen^™^). H_2_DCFDA stock of 10 m*M* was originally prepared in dimethyl sulfoxide (DMSO) and kept frozen in −20°C for no longer than a month before use. The assay plate was mixed thoroughly and analyzed at specific time intervals on a SpectraMax i3 multimode plate reader through the bottom reading technique, and the chamber was pre-warmed to 37°C. The parameters on the plate reader were set to λ_Exc/Emi_ = 500/529 nm, photomultiplier tube (PMT): medium, and 15 min interval.

### Immunoblot for protein expression

Protein analysis was performed *via* Western blotting on brain and kidney homogenates separated by sodium dodecyl sulfate-polyacrylamide gel electrophoresis and then transferred to a polyvinylidene difluoride membrane (Whatman) and blotted for CGL (ab8245; Abcam), CBS (ab135626; Abcam), glyceraldehyde 3-phosphate dehydrogenase (GAPDH; ab8245; Abcam), and αTubulin (ab4074; Abcam), followed by horseradish peroxidase (HRP)-conjugated secondary anti-rabbit antibody (ab97051; Abcam) or anti-mouse antibody (62-6520; Invitrogen). Proteins were visualized by using SuperSignal West Femto Maximum Sensitivity Substrate (No. 34096; Thermo Scientific) on an Amersham Imager 600 (General Electric), and size was determined by using the PageRuler Plus Prestained (No. 26619; Thermo Fisher).

### Real-time polymerase chain reaction for gene transcript expression

Gene transcript analysis was performed *via* real-time polymerase chain reaction (PCR) on brain samples of the Young Group mice. Total RNA was extracted by using TRIzol^™^ Reagent (No. 15596026; Invitrogen), reverse transcribed by using Verso complementary DNA (cDNA) synthesis kit (No. AB1453; Thermo Scientific), and quantified for each gene by using SYBR^™^ Green Master Mix reagent (No. 4385612; Applied Biosystems) on a StepOnePlus^™^ Real-Time PCR System (Thermo Scientific). The cDNA input ranged from 4 to 10 ng reverse-transcribed RNA depending on the target gene expression threshold. The expression of target genes was normalized to βActin and analyzed in fold changes by using the ΔΔCt method. Primer sequences for the genes of interest are as follows: *GST*α forward: 5′-CTGCAGCAGGGGTGGA-3′, *GST*α reverse: 5′-CCTTCCTGTACTTCCTCTCTC-3′; *TNF*α forward: 5′-CATCTTCTCAAAATTCGAGTGACAA-3′, *TNF*α reverse: 5′- TGGGAGTAGACAAGGTACAACCC-3′; nuclear factor kappa-light-chain-enhancer of activated B cells (*NF-*κ*B1a*) forward: 5′- GGAGACTCGTTCCTGCACTTGG-3′, NF-κB1a reverse: 5′- AACAAGAGCGAAACCAGGTCAGG-3′; interleukin 6 (*IL-6*) forward: 5′-TACCACTTCACAAGTCGGAGGC-3′, *IL-6* reverse: 5′-CTGCAAGTGCATCATCGTTGTTC-3′; interleukin 1 beta (*IL-1*β) forward: 5′-TGGACCTTCCAGGATGAGGACA-3′, *IL-1*β reverse: 5′-GTTCATCTCGGAGCCTGTAGTG-3′; heme oxygenase-1 (*HO-1*) forward: 5′-AAGCCGAGAATGCTGAGTTCA-3′, *HO-1* reverse: 5′-CGGGTGTAGATATGGTACAAGGA-3′; NAD(P)H Quinone Dehydrogenase-1 (*NQO-1*) forward: 5′-CAGATCCTGGAAGGATGGAA-3′, *NQO-1* reverse: 5′-TCTGGTTGTCAGCTGGAATG-3′.

### Peptide level iodoTMT-BTA

To identify and quantify persulfidated proteins, we used peptide level iodoTMT-biotin thiol assay followed by liquid chromatography–mass spectrometry (LC/MS) and pathway analysis recently described by our lab in Bithi et al. (5). Briefly, 7 mg of proteins was extracted and alkylated with Maleimide-PEG2-biotin. Alkylated proteins were precipitated with ice cold acetone, and they were suspended in denaturation buffer (8 M urea, 1 mM MgSO4 and 30 mM Tris–HCl, pH 7.5). After denaturation for 10 min at 90°C, proteins were diluted with 7 volumes of dilution buffer (1 mM CaCl2, 100 mM NaCl, and 30 mM HEPES-NaOH pH 7.5), followed by incubation with trypsin (No. V5111; Promega; ratio of 1:50 W/W) overnight at 37°C. After overnight digestion, trypsin was heat inactivated at 95°C for 10 min. Before adding the digested peptide to the streptavidin-agarose resin containing spin columns, the columns (Cat. No. 69705; Pierce) were first washed with PBS, followed by adding 0.400 mL streptavidin-agarose resin (No. 20347; Thermo Scientific) to the column and washing the resin 1 × with PBS. The resin was then equilibrated with a 1:7 denaturation buffer: dilution buffer, washed, and incubated at 4°C. After the overnight incubation, the protein resin mix was washed and eluted with 500 μL elution buffer (30 mM Hepes, 1 mM EDTA, 100 mM NaCl, 10 mM TCEP, 10 mM TEAB, pH 7.5). A C18 column (No. 89870; Thermo) was used to remove TCEP followed by eluting the peptides with 70% ACN. Eluted peptides containing thiol groups were alkylated and labeled with 2.95 mM iodoTMT (No. 90101; Thermo Scientific) for 1 h in the dark followed by quenching with 20 mM DTT. The alkylated peptides were mixed and combined. Each of the six samples labeled with a unique iodoTMT tag were then mixed into a single tube and dried. Finally, the combined labeled peptides were reconstituted with 1% Acetic Acid for further analyses via LC/MS.

### High-performance liquid chromatography-tandem mass spectrometric analysis

Digested peptides were analyzed on a ThermoFisher Scientific UltiMate 3000 high-performance liquid chromatography (HPLC) system (ThermoFisher Scientific, Bremen, Germany) interfaced with a ThermoFisher Scientific Orbitrap Fusion Lumos Tribrid mass spectrometer (Thermo Scientific, Bremen, Germany). Liquid chromatography was performed before tandem mass spectrometry (MS/MS) analysis for peptide separation. The HPLC column used is a Dionex 15 cm × 75 μm Acclaim Pepmap C18, 2 μm, 100 Å reversed-phase capillary chromatography column. Five microliter volumes of the peptide extract were injected; peptides were eluted from the column by a 90-min acetonitrile/0.1% formic acid gradient at a flow rate of 0.30 μL/min and introduced to the source of the mass spectrometer online. Nano electrospray ion source was operated at 2.3 kV. Data using the iodoTMT 6plex labeled peptide samples were collected by using a TMT-MS2 method in the same Orbitrap Fusion Lumos Tribrid mass spectrometer (Thermo Scientific). This was a data-dependent acquisition method using higher-energy collisional dissociation as the fragmentation method for MS/MS scans. It included one full scan followed by as many MS/MS scans as possible at collision energy 38 in a 3S duty cycle on the most abundant ions in that full scan. The detector of mass spectrometry (MS) full scans was the Orbitrap at a resolution of 120,000, and the detector of MS/MS scans was the Orbitrap at a resolution of 50,000. Dynamic exclusion was enabled with a repeat count of 1, and ions within 10 ppm of the fragmented mass were excluded for 60 s.

### Bioinformatics for peptide identification, quantification, and biological pathway enrichment

The MS raw data files of iodoTMT labeled peptides were analyzed by Proteome Discoverer version 2.4.1.15 (ThermoFisher Scientific) with the following settings. We used Mus musculus in SwissProt version 2019 (Swiss Institute of Bioinformatics) FASTA file. For the quantification method, we used iodoTMT6plex with the TMT reporter ion isotope distribution (lot number VH308730). For the processing setup, we used integration tolerance as 20 ppm and the integration method was set to the most confident centroid. The Fourier transform mass spectrometry mass analyzer was used, and we selected MS1 for the precursor selection section. For the enzyme name, we used trypsin (full) with a maximum missed cleavage site of 2. Precursor mass tolerance was set to 10 ppm, and fragment mass tolerance set to 0.2 Da. For dynamic modification, we allowed methionine oxidation (+15.995 Da). We selected static modification as iodoTMT6plex/+329.227 Da of cysteine. For the consensus setup, we used intensity as reporter abundance. Validation mode was set to automatic (control peptide level error rate if possible). Target false discovery rate (FDR; strict) for peptide-spectrum matches (PSMs) and target FDR (strict) for peptide were set at 0.01, whereas target FDR (relaxed) for PSMs and target FDR (relaxed) for peptide were set at 0.05. Peptide confidence was set to high with minimum peptide length allowed to 6. For the confidence threshold, we used target FDR (strict) and target FDR (relaxed) at 0.01 and 0.05, respectively. The minimum PSM confidence level was set to high. The online-based web server g:Profiler (https://biit.cs.ut.ee/gprofiler/gost) was used for functional enrichment analysis of identified persulfidated proteins. Gene ID's or accession number of enriched peptides/proteins were used in the g:GOSt (Gene Group Functional Profiling) identifier tool to detect biological pathways from the KEGG database significantly enriched with persulfidated proteins. These significance values were given a threshold of p < 0.05 and were auto-calculated by the software's proprietary g:SCS algorithm that utilizes multiple testing correction for p values gained from pathway enrichment analysis. In setting the boundaries for significance determination, it corresponds to an experiment-wide threshold of a = 0.05, with the g:SCS threshold pre-calculated for list sizes up to 1000 accession number or gene ID terms and analytically approximates a threshold t corresponding to the 5% upper quantile of randomly generated queries of that size. As per the g:Profiler description, all actual p values resulting from the query are automatically transformed to corrected p values by multiplying these to the ratio of the approximate threshold t and the initial experiment-wide threshold a = 0.05 with consideration to the underlying gene sets annotated to terms from the KEGG database, and therefore gives a tighter threshold to significant results and is more conservative than the Benjamini-Hochberg FDR.

### Statistical analysis

Data are displayed as means ± standard error of the mean, with n values indicated in the figure legends. An AUC was calculated with baseline set at 0 unless stated elsewhere and depicted as in-set charts within the figure panel. Statistical significance was assessed in GraphPad Prism 8 software (GraphPad Software, Inc., La Jolla, CA), with the methods described in the following paragraphs.

For body weight and body composition analyses, body weight was converted to the percentage to individual initial value at 5 weeks of age. The ratios of body composition to individual BM, for LM (LM/BM) and FM (FM/BM), were also converted to the percentage of its ratio at initial measurement at 5 weeks of age for the Young Group and 26 weeks of age for the Aged Group animals. To compare the variable (BPA) over a time course, including the measurement of body weight, body composition, and food intake, pairwise comparison of means was performed by using the repeated-measures mixed model ANOVA, with terms for BPA, age, and their interaction. Notably, although ANOVA is used when one of the factors is quantitative, such as age in this study, it does not factor in the order of the time points. Therefore, the AUC was calculated for each group, followed by a one-way ANOVA analysis with Dunnett multiple-comparison tests. The baseline of AUC analysis for body weight was set at 100. Unlike two-way ANOVA, this kind of analysis follows the scientific logic of the experiment, which is to evaluate the overall measure of cumulative response. A similar one-way ANOVA analysis of AUC was performed for the GTT and the baseline of AUC analysis was to the mean of initial fasting glucose concentrations for each dose-defined group.

For single end-point assessment of the variable, including fasting glucose level, serum IGF-1, serum tT4 levels, H_2_S production capacity, immune-blots, and REDOX measurements, unless specified in the figure legend, one-way ANOVA was performed with Dunnett multiple-comparison tests. The derived *p* value <0.05 was considered statistically significant and annotated with a signal in the figure and figure legend.

In the OF test, the AUC was calculated for each group for the analyses of distance moved and duration in the inner area data; this was followed by a two-way ANOVA with Dunnett multiple-comparison tests that was performed to test the effects of Age and BPA. Other measurements without AUC calculation were also analyzed by using the two-way ANOVA (Age × BPA) method. The derived *p* value <0.05 was considered statistically significant and annotated as the star (*) signal. In the following novel object location recognition test, paired two-tail *t*-tests were performed to compare the explorative difference between the moved object and unmoved object within the same BPA dose group in the specific measurement. The derived *p* value <0.05 was considered statistically significant and annotated as the asterisk (*) signal. To test the effect of BPA on spatial memory, a two-way ANOVA with Dunnett multiple-comparison tests was used to compare the DI of duration for both for age-defined group animals. The derived *p* value <0.05 was considered statistically significant and annotated as the plus (+) signal. In the Y-maze test, to test the effect of BPA on the explorative activity, paired two-tail *t*-tests were performed to compare the measurement in two arms. The derived *p* value <0.05 was considered statistically significant. The star (*) sign indicates the significance between the start arm and known arm, and the number (#) sign annotates the significance between the target arm and known arm. To test the effect of BPA on spatial memory, a two-way ANOVA with Dunnett multiple-comparison tests was used to compare the duration, the preference score, the frequency, as well as the percent frequency to the target arm for age-defined group animals. The derived *p* value <0.05 was considered statistically significant and annotated as the plus (+) signal.

Data were deposited into folders and servers backed on the Cleveland Clinic Lerner Research Institute Network, with proteomic data uploaded to ProteomeXchange Consortium *via* PRIDE repository with Project Accession Number: PXD025746 and Project Webpage: www.ebi.ac.uk/pride/archive/projects/PXD025746

## Data Availability Statement

The data supporting the claims in the article are available in the Figures and Supplementary Figures and data files. In addition, raw data including behavior video data are available on request to the corresponding author. Proteomics data are uploaded to the ProteomeXchange Consortium *via* PRIDE repository with Project Accession number: PXD025746 and Project Webpage: www.ebi.ac.uk/pride/archive/projects/PXD025746

## Supplementary Material

Supplemental data

Supplemental data

Supplemental data

Supplemental data

Supplemental data

Supplemental data

Supplemental data

Supplemental data

Supplemental data

Supplemental data

Supplemental data

Supplemental data

Supplemental data

Supplemental data

Supplemental data

## Supplementary Material

YangSupplementalFigures

YangUncroppedBlotsAndImagesFIG6andSupplementalFIG6

Fig 6A Statistics

YangSourceBrainPersulfidationDataFigure6

YangSourceDataFIGURE2

YangSourceDataFIGURE3

YangSourceDataFIGURE4

YangSourceDataFIGURE5

YangSourceDataFIGURE6

YangSourceDataFIGURE7andSupplementalFIGURE7

YangSourceDataSupplementalFigure2

YangSourceDataSupplementalFigure3

YangSourceDataSupplementalFigure4

YangSourceDataSupplementalFigure5

YangSourceDataSupplementalFigure6

## Abbreviations Used

ANOVA: analysis of variance
AUC: area-under-the-curve
BM: body mass
BPA: bisphenol A
b.w.: body weight
CBS: cystathionine β-synthase
cDNA: complementary DNA
CGL: cystathionine γ-lyase
CLARITY-BPA: Consortium Linking Academic and Regulatory Insights on Bisphenol A Toxicity
CTL: control
DHR123: dihydrorhodamine 123
DI: discrimination index
Dur: duration
EDC: endocrine disrupting chemical
ER: estrogen receptor
FDR: false discovery rate
FM: fat mass
Freq: frequency
GAPDH: glyceraldehyde 3-phosphate dehydrogenase
GD: gestational day
GST: glutathione S-transferase
GTT: glucose tolerance test
H_2_DCFDA: 2′,7′-dichlorofluorescin diacetate
H_2_S: hydrogen sulfide
HD: high dose
HO-1: heme oxygenase-1
IGF-1: insulin-like growth factor 1
IL-1β: interleukin 1 beta
IL-6: interleukin 6
LD: low dose
LM: lean mass
LOAEL: lowest-observed-adverse-effect-level
NF-κB1a: nuclear factor kappa-light-chain-enhancer of activated B cells
NLR: novel object location recognition task
NQO-1: NAD(P)H quinone dehydrogenase-1
OF: open field
PBS: phosphate buffered saline
PCR: polymerase chain reaction
PND: postnatal day
qPCR: quantitative polymerase chain reaction
RT: retention trial
SEM: standard error of the mean
ST: sample trial
TH: thyroid hormone (T3 and T4)
TNFα: tumor necrosis factor alpha
tT4: total thyroxine (thyroid hormone)
