# Supplementary material for: Peripubertal Bisphenol A Exposure Imparts Detrimental Age-Related Changes in Body Composition, Cognition, and Hydrogen Sulfide Production Capacities
Source: Antioxid Redox Signal. 2022 Jun 3;36(16-18):1246–67. doi: 10.1089/ars.2020.8226 (PMC9221154; doi:10.1089/ars.2020.8226)
Supplement: Supplemental data [file Supp_Figures.pdf]

Supplemental Figures and Legends

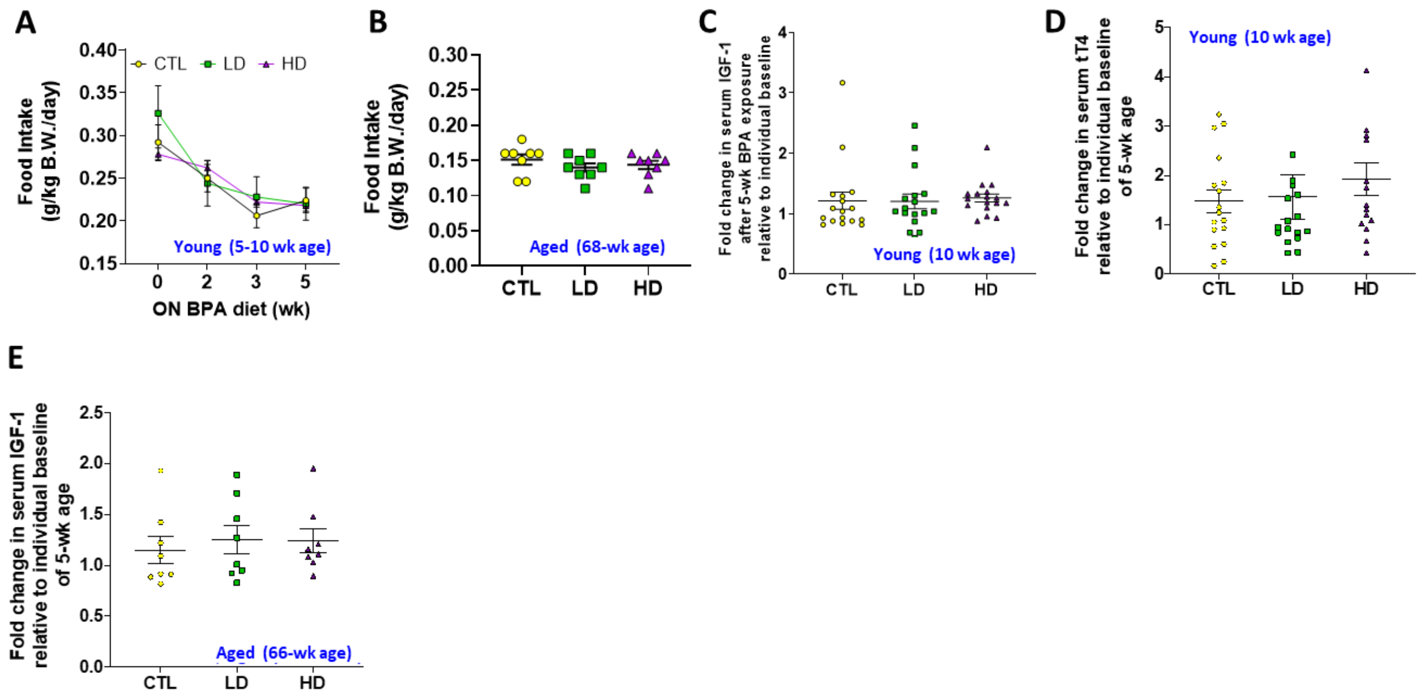

Supplemental Figure 2: Food intake, hormonal changes and fasting blood glucose of male mice during or post BPA experimental diets. (**A-B**): Food intake of the Young Group animals (**A**) during BPA experimental diets and the Aged Group animals (**B**) at 68 weeks of age were assessed and presented as daily food intake per kilogram body weight. n = 5/group in the Young Group and 8/group in the Aged Group. (**C-E**): Serum IGF-1 (**C**) and total T4 (tT4; **D**) were assayed in Young Group animals (n = 17/group) at 10 weeks of age and (**E**) IGF-1 in Aged Group animals at 66 weeks of age (n = 8/group) and presented as the fold changes relative to the respective individual baseline levels at 5 weeks of age. All data presented as mean ± SEM. *See also Figure 2*.

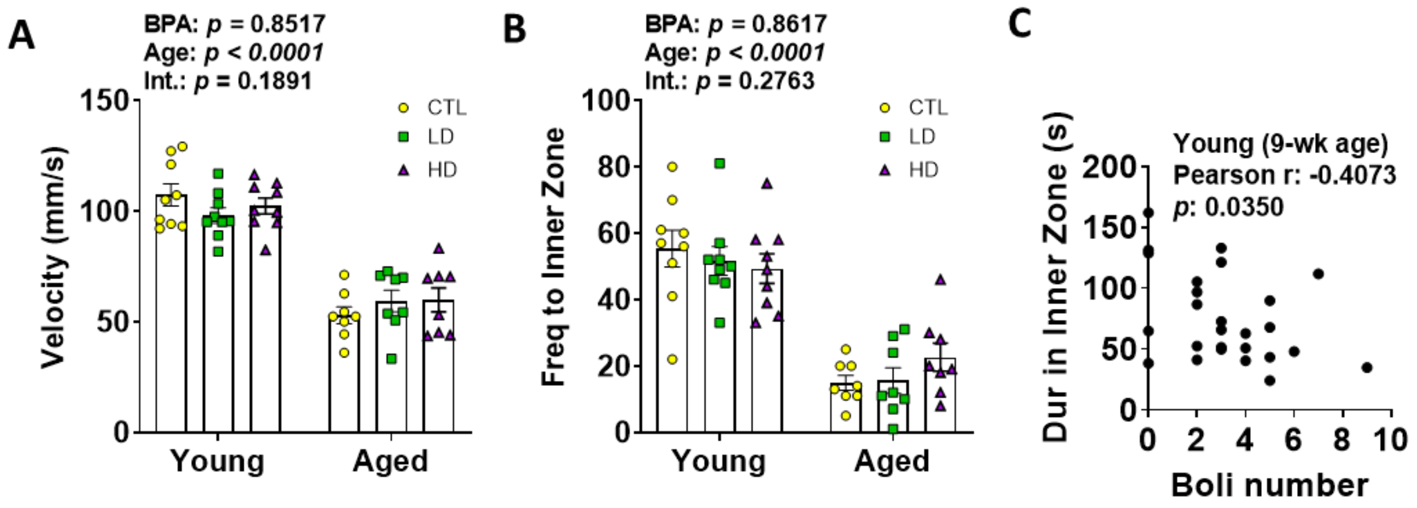

Supplemental Figure 3: Validity of the open field test to detect experimental variables of velocity and frequency in the inner zone comparing Young and Aged mice. (**A-B**): Velocity (**A**) and frequency to the inner zone (**B**) of the Young and Aged Group animals peripubertally exposed to BPA experimental diets, n = 8-9/group. (**C**): Validating markers of anxiety via correlation analysis between time spent in the inner zone and feces defecation in the Young Group animals, n = 8-9/group. *See also Figure 3*.

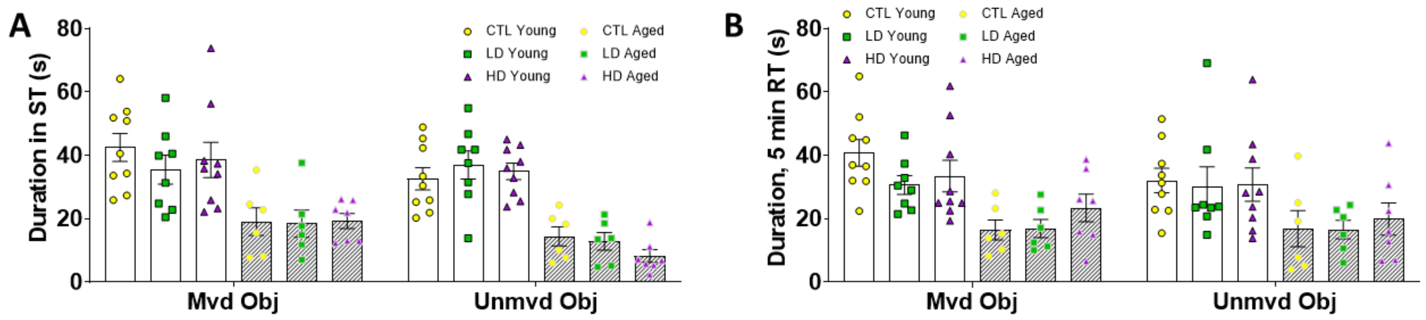

Supplemental Figure 4: Duration at both objects in novel object location recognition test for Sample Trial (ST) and Retention Trial (RT). (**A**): Duration at both objects in total 5-minute ST. (**B**): Duration at both objects in total 5-minute RT. Clear bars are the Young Group and shadowed bars are the Aged Group. n = 8-9/group in the Young Group and 6-7/group in the Aged Group. All data were presented as mean ± SEM. *See also Figure 4*.

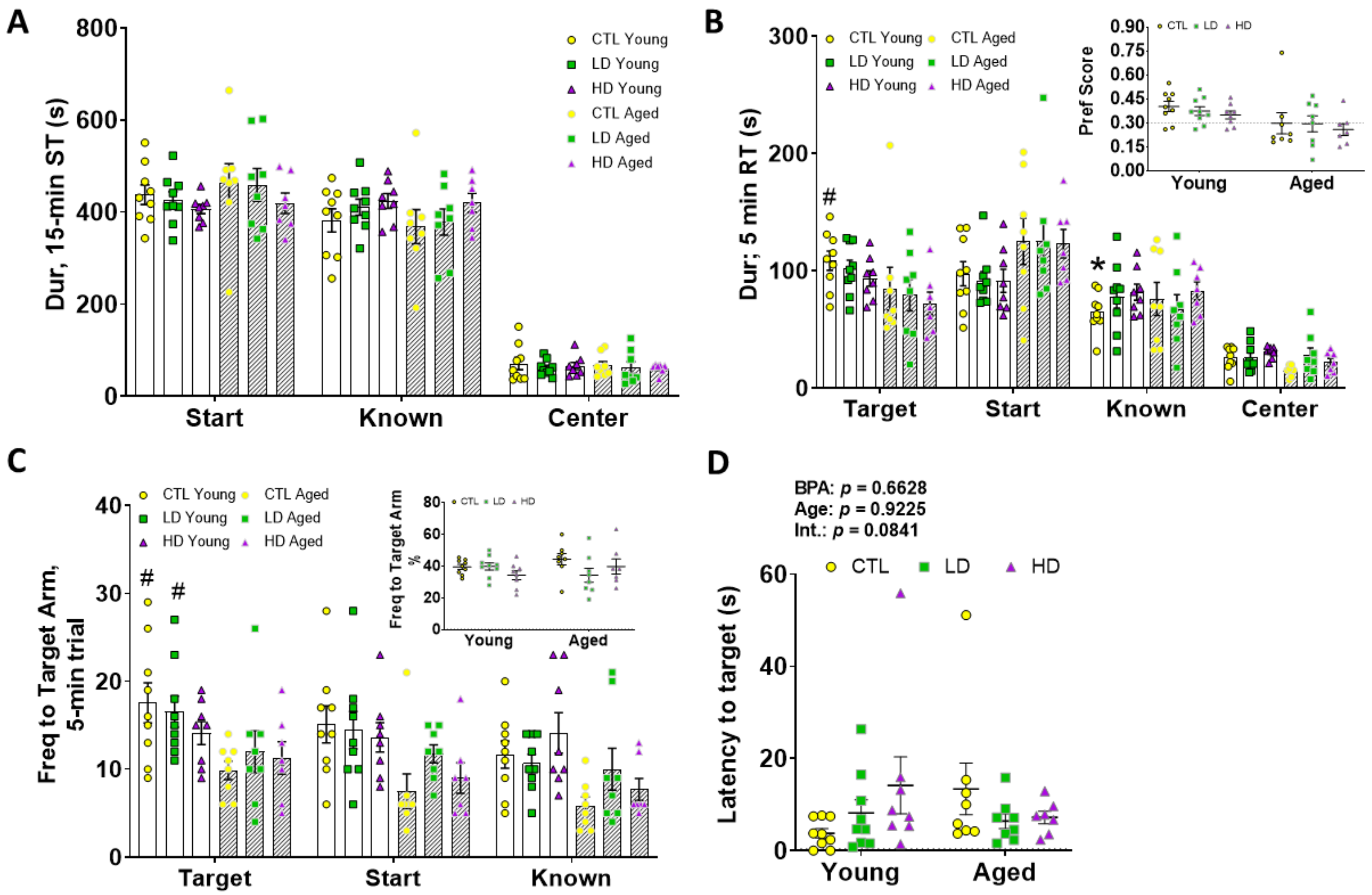

Supplemental Figure 5: Y-maze spatial learning and memory tests. (**A**): Duration in each arm and the center during the total 15-minute sample trial (ST). (**B-C**): Duration in each arm and the center (**B**) and frequency to each arm (**C**) during the total 5-minute retention trial (RT). The preference score and percent frequency to the target arm were depicted as in-sets to the figure. (**D**): Latency to the target arm in the RT. Clear bars are the Young Group and shadowed bars are the Aged Group. n = 8-9/group in the Young Group and 7-8/group in the Aged Group. Asterisk indicates the significance derived from paired *t*-test of performance in known arm versus start arm; * $P < 0.05$. Number sign indicates the significance derived from paired *t*-test of performance in target arm versus known arm; # $P < 0.05$. All data were presented as mean ± SEM. *See also Figure 5*.

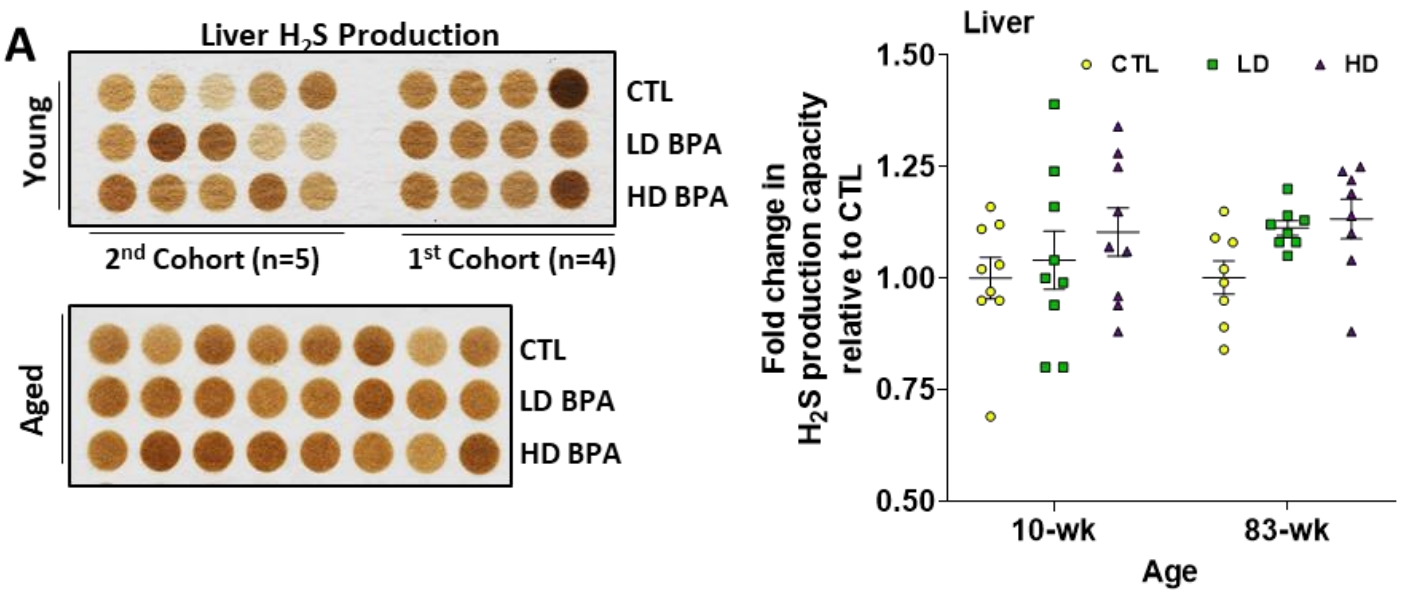

Supplemental Figure 6: Peripubertal BPA exposure did not alter immediate or late-life hepatic H_2S production capacity. (**A**): H_2S production capacity in the liver of the Young and Aged Group animals. n = 9/group in the Young Group and 8/group in the Aged Group. All data were presented as mean ± SEM. *See also Figure 6*.

A

Young Group CTL+LD+HD			Renal H_2S	Brain H_2S	Physiology		Anxiety		Hippocampal-Dependent (HD) Memory				Oxidative Stress				H_2S Producing Genes		
					Body composition		Open Field		Y-Maze				Kidney		Brain		Kidney		Brain
					Lean Mass %	Fat Mass %	Duration in Inner	Latency to Inner	Duration in Target	Preference Score	Frequency to Target	Frequency to Target %	DCFHDA	DHR123	DCFHDA	DHR123	CGL	CBS	CBS
		Renal H_2S	1.00																
		Brain H_2S	*0.53*	1.00															
		Lean Mass %	0.03		1.00														
		Fat Mass %	-0.08		**-0.95**	1.00													
Anxiety	Open Field	Duration in Inner	-0.11	-0.05	0.00	0.19	1.00												
Anxiety	Open Field	Latency to Inner	-0.22	*-0.52*	**-0.59**	0.55	*-0.35*	1.00											
HD Memory	Y-Maze	Duration in Target	0.19	0.16	0.23	-0.36	0.29	-0.19	1.00										
HD Memory	Y-Maze	Preference Score	0.22	0.15	0.19	-0.31	0.30	-0.17	**0.99**	1.00									
HD Memory	Y-Maze	Frequency to Target	**0.39**	0.20	-0.12	-0.05	0.27	-0.32	**0.59**	**0.63**	1.00								
HD Memory	Y-Maze	Frequency to Target %	**0.48**	0.35	-0.13	-0.07	0.04	-0.32	**0.58**	**0.60**	**0.72**	1.00							
Oxidative stress	Kidney	DCFHDA	-0.07	-0.25	0.30	-0.26	0.09	-0.02	-0.20	-0.21	-0.18	0.03	1.00						
Oxidative stress	Kidney	DHR123	-0.19	-0.10	0.25	-0.17	-0.11	0.07	-0.19	-0.21	**-0.57**	-0.34	0.07	1.00					
Oxidative stress	DCFHDA	Cellular	-0.34	-0.05			0.24	-0.36	-0.41	-0.43	-0.30	-0.23	0.21	0.12	1.00				
Oxidative stress	DCFHDA	DHR123I	-0.32	-0.37			0.37	-0.25	0.01	0.00	-0.28	0.01	0.21	0.41	**0.67**	1.00			
H_2S producing genes	Kidney	CGL	0.06		-0.27	0.27	0.00	0.31	-0.42	-0.38	-0.04	0.09	-0.18	0.21			1.00		
H_2S producing genes	Kidney	CBS	0.15		-0.31	0.16	0.17	0.06	-0.06	-0.05	0.28	0.33	0.02	-0.18			0.34	1.00	
H_2S producing genes	Brain	CBS	0.43	-0.10			0.37	0.21	0.49	*0.50*	0.18	0.14	-0.15	0.07	-0.25	0.24			1.00

Pearson Correlation r

-1.0 -0.5 0 0.5. 1.0

Bold r: $p < 0.05$ (two-tailed)
Italic underlined r: $0.05 < p < 0.1$ (two-tailed)

B

Aged Group CTL+LD+HD			Renal H2S	Brain H2S	Physiology and Glucose Metabolism			Anxiety		Cognition			
					Body composition		Fasting blood glucose	Open Field		Y-Maze			
					Lean Mass %	Fat Mass %		Duration in Inner	Latency to Inner	Duration in Target	Preference Score	Frequency to Target	Frequency to Target %
		Renal H2S	1.00										
		Brain H2S	0.15	1.00									
		Lean Mass %	-0.02	**0.58**	1.00								
		Fat Mass %	0.12	**-0.47**	**-0.94**	1.00							
		Fasting blood glucose	0.33	*-0.51*	*-0.55*	0.35	1.00						
Anxiety	Open Field	Duration in Inner	-0.05	0.25	*0.35*	**-0.47**	**-0.61**	1.00					
Anxiety	Open Field	Latency to Inner	-0.03	-0.04	-0.32	0.26	0.13	-0.32	1.00				
Cognition	Y-Maze	Duration in Target	-0.03	-0.02	-0.02	0.09	0.14	0.01	*-0.39*	1.00			
Cognition	Y-Maze	Preference Score	-0.03	0.02	0.03	0.05	0.16	-0.02	*-0.40*	**0.99**	1.00		
Cognition	Y-Maze	Frequency to Target	-0.29	*0.40*	**0.44**	-0.29	-0.26	-0.07	-0.21	**0.63**	**0.68**	1.00	
Cognition	Y-Maze	Frequency to Target %	-0.01	0.09	0.18	-0.02	0.10	-0.21	*-0.36*	**0.63**	**0.64**	**0.73**	1.00

Pearson Correlation r

-1.0 -0.5 0 0.5. 1.0

Bold r: $p < 0.05$ (two-tailed)
Italic underlined r: $0.05 < p < 0.1$ (two-tailed)

Supplemental Figure 7: Pearson correlation analysis between variables as indicated in the Young (**A**) and Aged (**B**) Group mice peripubertally fed with Control and LD and HD BPA containing experimental diets. n = 15-27/group in the Young Group and 23-24/group in the Aged Group. Pearson correlation coefficients also referred to as *r* values between two variables were annotated in the box. Red color indicates positive correlations and blue color indicates negative correlations, with their intensity being proportional to the *r* values. The color legend in the right side of the chart indicates the *r* values and the corresponding colors. Bolded number indicate reaching statistical significance with $p < 0.05$, while underlined italic numbers indicating approaching statistical significance with $0.05 < p < 0.1$. *See also Figure 7.*
